# The Influence of Microwave-Assisted Extraction on the Phenolic Compound Profile and Biological Activities of Extracts from Selected *Scutellaria* Species

**DOI:** 10.3390/molecules28093877

**Published:** 2023-05-04

**Authors:** Joanna Oracz, Stanisław Kowalski, Dorota Żyżelewicz, Gabriela Kowalska, Dorota Gumul, Kamila Kulbat-Warycha, Justyna Rosicka-Kaczmarek, Anna Brzozowska, Aleksandra Grzegorczyk, Anna Areczuk

**Affiliations:** 1 Institute of Food Technology and Analysis, Faculty of Biotechnology and Food Sciences, Lodz University of Technology, 2/22 Stefanowskiego Street, 90-537 Lodz, Poland; gabriela.kowalska@p.lodz.pl (G.K.); kamila.kulbat-warycha@p.lodz.pl (K.K.-W.); justyna.rosicka-kaczmarek@p.lodz.pl (J.R.-K.);; 2 Department of Carbohydrate Technology and Cereal Processing, Faculty of Food Technology, University of Agriculture in Krakow, Balicka Str. 122, 30-149 Krakow, Poland; rrkowals@cyf-kr.edu.pl (S.K.); rrgumul@cyf-kr.edu.pl (D.G.); anna.areczuk@urk.edu.pl (A.A.)

**Keywords:** *Scutellaria baicalensis*, *Scutellaria lateriflora*, phenolic compounds, flavonoids, antioxidant activity, anti-inflammatory activity

## Abstract

The aim of the study was to investigate the effects of microwave-assisted extraction (MAE) conditions (microwave power, extraction time, and ethanol concentration) on the efficiency of the extraction of phenolic compounds from selected plant species belonging to the genus *Scutellaria* (i.e., *Scutellaria baicalensis* and *Scutellaria lateriflora*). The extracts from selected *Scutellaria* species were examined to establish the total phenolic content and the in vitro antioxidant and anti-inflammatory activity. The antioxidant capacity was determined by the ferric reducing antioxidant power (FRAP) and 2,2,1-diphenyl-1-picrylhydrazyl (DPPH) radical scavenging capacity methods. The anti-inflammatory activity was evaluated through the lipoxygenase (LOX) inhibitory assay. The phenolic profile of the extracts was characterized using ultra-high performance liquid chromatography coupled with diode array detection and high-resolution electrospray ionization mass spectrometry (UHPLC–DAD/ESI–HRMS/MS). Depending on the type of solvent and the extraction conditions used, the extracts obtained from selected *Scutellaria* species showed different total and individual phenolic content, as well as different antioxidant and anti-inflammatory properties. The results showed that all *Scutellaria* extracts had high total phenolic content and exhibited strong ferric ion reducing power and free radical scavenging capacity and a significant ability to inhibit the LOX activity. In general, the 70% ethanol extracts contained more phenolic compounds, mainly flavones, flavanones, and their derivatives, and showed greater in vitro biological activity than other extracts. The highest levels of phenolic compounds and the strongest antioxidant and anti-inflammatory potential were found in extracts from the roots of *S. baicalensis*. Optimal extraction conditions for all the plant materials tested were determined as the microwave power of 63 W, extraction time of 10 min, and 70% ethanol as the solvent.

## 1. Introduction

Plants of the genus *Scutellaria* belonging to the *Lamiaceae* family are cosmopolitan and are present across different continents with different climatic zones, mainly in North America, Europe, and East Asia. Species belonging to the genus *Scutellaria* L. are annual or perennial herbaceous plants [1,2,3]. *Scutellaria baicalensis* Georgi (known as Chinese Skullcap or Baikal Skullcap), due to its therapeutic properties, is the best-known species in the genus *Scutellaria* and has been used in Chinese medicine for over two millennia. In the Chinese Pharmacopoeia, this plant is called Huang-Qin. This plant originally comes from Asia, growing mainly in a dry mountain climate in China and Russia (near Lake Baikal), as well as Mongolia, Japan, and Korea [4,5].

Species of the genus *Scutellaria* L. have been used in phytotherapy for centuries. The metabolites isolated from *Scutellaria* total over 2000, of which around 800 are of potential medical importance [6]. Among all of the above, flavonoids are considered the largest group of compounds present in these plants that affect their phototherapeutic activity.

The *S. baicalensis* plant contains flavonoid glycosides, which include, e.g., baicalin, wogonoside, dihydrobaicalin, norwogonin [7], and scutellarin [8]. The first two of the listed components occur as dominant compounds in the roots of *S. baicalensis*, while scutellarin is found in higher concentrations in stems and leaves [9]. Among flavonoids in the form of aglycones, such flavones as baicalein, oroxylin A, wogonin, chrysin, scutellarein, neobaicalein, and skullcapflavone II have been detected. Three sterols have been also identified in the roots of *S. baicalensis* extracts, namely β-sitosterol, stigmasterol, and daucosterol [7].

Due to the presence of various flavonoids, plants of the genus *Scutellaria* L. exhibit biological activity against various microorganisms. The root of *S. baicalensis* has been used in Chinese medicine in the treatment of different infections. In vitro tests have proven the fungicidal effectiveness of aqueous extracts against *Aspergillus fumigatus*, *Candida albicans*, *Geotrichum candidum*, and *Rhodotorula rubra* [10], as well as dermatophytes *Trichophyton rubrum* and *Trichophyton mentagrophytes* [11]. The flavonoid responsible for the antifungal properties is baicalein. The beneficial effect is possible due to the specific structure of this compound. Furthermore, baicalein and baicalin are considered to be the main compounds of *S. baicalensis* that exhibit antibacterial activity. In vitro studies have proven their activity against such types of bacteria as *Escherichia coli*, *Bacillus cereus*, *Staphylococcus aureus*, *Listeria monocytogenes*, and *Salmonella anatum* [12]. Additionally, Chinese Skullcap extract, due to its properties, exhibits a synergistic effect together with β-lactam antibiotics, i.e., it strengthens their activity against *Staphylococcus aureus* [13].

The root of *S. baicalensis* is rich in flavonoids with high antioxidant activity; baicalein is characterized by the greatest ability to neutralize reactive oxygen species, followed by baicalin, wogonin, and wogonoside. It should be emphasized that the radical scavenging activity of wogonin and wogonoside is low compared to the dominant baicalein [14]. Flavonoids isolated from *S. baicalensis* roots act also as anti-inflammatory and antiallergic agents. Flavonoids, including baicalein, baicalin, wogonin, wogonoside, and oroxylin A, can have a direct effect on the cells of the immune system and inhibit the secretion of inflammatory mediators such as cytokines IL-1β, IL-6, IL-8, and TNF-α, as well as prostaglandins and leukotrienes. The ethanol extract and baicalein and wogonin alone can also reduce inflammation by inhibiting the number of eosinophils and the release of histamine and leukotrienes from mast cells, which was confirmed in an animal model in mice. Considering the above, baicalein and wogonin may have therapeutic potential in allergic diseases, including asthma [15,16]. Due to the wide spectrum of properties, especially anti-inflammatory, *S. baicalensis* may also be used in the treatment of autoimmune diseases [16,17], but this requires further research.

Prolonged inflammation is one of the factors leading to the development of neoplastic changes. Baikal skullcap flavonoids have the ability to reduce the concentration of pro-inflammatory cytokines and thus reduce the risk of permanent inflammation, protecting against cancerogenesis. It has also been proven that wogonin is an excellent anti-inflammatory agent that does not cause numerous side effects, as is the case with other drugs with such an effect. In addition, in vitro, wogonin induced the apoptosis of pathologically changed cells, which was confirmed in human osteosarcoma cells [18], human breast cancer [19], human glioma cancer [20], and even HL-60 leukemia cells [21].

A new area of research on the properties of *S. baicalensis* is its anxiolytic and antidepressant properties. In traditional Chinese medicine, it is considered a powerful anti-stress agent. It has also been proven that the root extract has anticonvulsant and spasmolytic properties. This effect is mainly due to baicalin, which is an agonist of gamma-amino butyric acid (GABA) receptors. Further, baicalin promotes hippocampal neurogenesis and the differentiation of neural stem/progenitor cells, which was confirmed in an animal model [22,23,24]. Scientific reports suggest that baicalin, due to its bioactive properties, including the inhibition of oxidative stress, excitatory poisoning, apoptosis, and inflammatory, stimulates neurogenesis and promotes the expression of neuron protective factors, which may be helpful in the treatment of neurological diseases such as ischemic stroke, Alzheimer’s, Parkinson’s, and Huntington’s diseases [25,26].

Besides *S. baicalensis*, the most valuable species of the genus *Scutellaria* is *Scutellaria lateriflora* (American skullcap, blue skullcap). This species has a long history of use in traditional medicine by the indigenous people of North America. The herb *S. lateriflora* is used as a sedative and anticonvulsant in the treatment of epilepsy. It supports the nervous system, reduces anxiety and restlessness, and prevents insomnia and various types of spasms [27,28]. The main phenolic compounds of *S. lateriflora* are flavonoids, including baicalein, wogonin, scutellarein, oroxylin A, baicalin, wogonoside, and scutellarin [27,28].

Due to the various concentrations of some of these bioactive compounds in the different species of the genus *Scutellaria*, it is essential to obtain the maximum yield of phenolic compounds by means of an extraction process that is reproducible, time-saving, and ecological. The choice of extraction method should take into account the thermolability of the bioactive compounds, and therefore the temperature and other parameters should be optimized. For the process to be effective, it is also necessary to consider the sample matrix and the type and location of the bioactive compounds within the matrix. Several authors have investigated various extraction techniques to isolate bioactive compounds from *Scutellaria* plants [29,30,31,32,33]. For example, Zhou et al., (2016) [33] used heat reflux extraction (HRE) with water at 100 °C for 2 h, ultrasound-assisted extraction (UAE) with water and 70% ethanol at 45 °C for 1 h, with the same ultrasonic frequency (40 kHz) and power (250 W), and microwave-assisted extraction (MAE) with water (at 100 °C for 2 h at 300 W) to extract phenolic compounds from the dried roots of *S. baicalensis* Georgi. They found that among all the extraction techniques, the most efficient method for extraction based on the yield of the crude herbal extract was MAE with water, followed by UAE with 70% ethanol, HRE, and UAE with water [33]. Zhang et al. (2015) [30] investigated the influence of different extraction methods, including MAE using water or ionic liquids (80 °C, 400 W for 90 s), HRE using water or ionic liquids (80 °C for 30 min), and the pharmacopeial method (70% ethanol, 80 °C and reflux for 3 h), on the content of bioactive compounds including flavonoids in the extracts obtained from *S. baicalensis* roots. These authors demonstrated that MAE using ionic liquid as an extraction solvent was the most effective method for the extraction of flavonoids, such as baicalin, wogonoside, baicalein, and wogonin [30].

Based on the data available in the literature [30,31,33], it can be assumed that microwave-assisted extraction (MAE) may be the optimal method for the extraction of phenolic compounds from *Scutellaria* plants. The results of previous studies indicate that MAE has great advantages in the extraction of bioactive compounds from *Scutellaria* spp. plants, such as the highest yield of crude extract, the shortest extraction time, and less solvent consumption when compared to conventional extraction methods [30,33]. Recently, this modern extraction technique has become more popular and common. It is a green and safe technology for extracting bioactive compounds using only water or ethanol as a solvent. Moreover, MAE is considered to be a more efficient technique for the extraction of phenolic compounds and even essential oils than conventional methods [34,35,36,37]. The higher efficiency of extraction of phenolic compounds from the plant matrix by MAE may be due to the faster and greater damage to the cell wall structure of the plant material as a result of the combination of a high temperature and microwave radiation, which is converted into heat by ionic conduction or dipole rotation, so that heat is generated directly in the plant tissue. In addition, microwave heating creates significant pressure within the plant matrix. This improves the porosity of the cell structure, allowing the extraction solvent to penetrate more easily and facilitating extraction by various methods using water or ethanol for the solvent diffusion of compounds from the plant matrix [38]. As a result, MAE allows for effective extraction in a very short time, using only a small amount of solvent [29,30,31,32,33,34,35,36,37,38].

However, for high-quality extracts, not only the extraction method but also its parameters are very important [33,37,38,39,40]. Generally, organic solvents (methanol and ethanol), water, and their mixtures have been widely used to isolate phenolic compounds, including flavonoids, from various plants using MAE. The efficiency of the MAE process depends mainly on the microwave power and the type of solvent, which must be a polar compound such as water, methanol, ethanol, and their mixtures [37]. According to Ni et al. (2018) [39], the solid–liquid ratio also plays a crucial role in attaining the maximum extraction yield of baicalin. Although previous studies have reported the effects of different extraction methods on the phytochemicals and bioactivity of *S. baicalensis* root extracts, no studies have determined the effects of different MAE conditions (the type of solvent, irradiation time, microwave power) on the total phenolic content, antioxidant activity, and inhibitory effect on lipoxygenase activity, as well as the extraction efficiency of phenolic compounds from the aerial parts of *S. baicalensis* and *S. lateriflora*.

Therefore, the aim of this study was to evaluate and optimize the MAE process of phenolic compounds (mainly flavonoids) from selected species belonging to the genus *Scutellaria* (i.e., *S. baicalensis* and *S. lateriflora*), and to investigate the qualitative and quantitative compositions of the phenolic compounds and the bioactivity (antioxidant and anti-inflammatory) of the obtained extracts. The optimization of the extraction process included the selection of the extraction solvent and the determination of the effect of the time and the power of the microwave radiation on the extraction efficiency of phenolic compounds and the antioxidant properties of the obtained extracts. Different microwave powers (21, 42, and 63 W) and extraction times (5 and 10 min) and three extractants (water, 40% ethanol, and 70% ethanol) in different combinations were used. The total phenolic content of the extracts was determined using the Folin–Ciocalteu assay. The antioxidant activity was evaluated using different in vitro tests, including 2,2,1-diphenyl-1-picrylhydrazyl (DPPH) radical scavenging and ferric reducing antioxidant power (FRAP). The in vitro anti-inflammatory activity of the extracts was determined by investigating the inhibition of the lipoxygenase (LOX) enzyme. The profile of phenolic compounds in extracts of dried roots and leaves of *S. baicalensis* and dried leaves of *S. lateriflora* was determined using ultra-high performance liquid chromatography coupled with diode array detection and high-resolution electrospray ionization mass spectrometry (UHPLC–DAD/ESI–HRMS/MS).

## 2. Results and Discussion

### 2.1. The Extraction Yield and Total Phenolic Content of the Extracts of Selected Species of Scutellaria

This is the first study that has attempted to determine the effects of different MAE conditions on the profile of phenolic compounds and the bioactivity of extracts from the roots and leaves of *S. baicalensis* and leaves of *S. lateriflora*. In order to evaluate the efficiency and optimize the extraction of phenolic compounds from the underground and aerial parts of selected *Scutellaria* spp. Using MAE, different solvents (water, 40 and 70% ethanol), microwave powers (21, 42, and 63 W), and extraction times (5 and 10 min) were used. Water is a widely used extraction solvent because it is non-toxic, non-flammable, readily available, and environmentally friendly, and its physicochemical properties change with temperature and pressure [41]. However, several studies have shown that methanol and ethanol are the most efficient organic solvents for the extraction of flavonoids from *Scutellaria* spp. plants [42]. Since methanol is highly toxic and not suitable for food and pharmaceutical processing, ethanol is much more commonly used for the extraction of bioactive compounds, such as flavonoids, from plant materials because of its effectiveness and safety [38]. Ethanol and ethanol–water solutions are less toxic and more sustainable than other organic solvents, but they can also have harmful biological effects on the human body. However, ethanol can be easily recovered by distillation under reduced pressure. Previous studies have also shown that the extraction efficiency of flavonoids from the roots and leaves of plants belonging to *Scutellaria* spp. can be improved by replacing water and pure ethanol with a mixture of ethanol with water at concentrations of 40–50% (*v/v*) [32] and 60–70% (*v/v*) [43]. Thus, in the current study, water and 40 and 70% ethanol were used as extraction solvents. The extraction yield of phenolic compounds from plant materials is also significantly influenced by the solid to solvent ratio [39]. Generally, a higher solvent/solid ratio is required to achieve a higher extraction yield of phenolic compounds. However, if the solvent to solid ratio is too high, excessive extraction solvent is consumed, requiring longer extraction times [39]. Therefore, based on previous studies [44], a 1:20 (*w/v*) ratio of dried plant tissue to solvent was chosen.

The influence of the extraction parameters, such as the microwave power, time of irradiation, and solvent, on the extraction yield are shown in Table 1. The results showed that the MAE conditions had significant effects on the extraction yields of phenolic compounds from different *Scutellaria* spp. plant materials. The crude extract yield ranged from 249.36 mg/g to 373.47 mg/g for dried leaves of *S. baicalensis*, from 279.77 mg/g to 386.05 mg/g for dried leaves of *S. lateriflora*, and from 349.16 to 488.03 mg/g for dried roots of *S. baicalensis*. Regardless of the plant material, the highest yields were observed for extracts obtained at the highest microwave power and a longer extraction time. It was found that increasing the ethanol concentration up to 70% increased the extraction yield. These results are consistent with other reported results [30,33,43,45].

Other authors also showed that the extraction of *S. baicalensis* roots using water with ethanol concentrations of 60–70% (*v/v*) allows higher yields of phenolic compounds to be obtained than when using water or ethanol as a solvent [43]. In the present study, the optimal MAE conditions for the maximum crude extract yields from different *Scutellaria* spp. were microwave power of 63 W, an irradiation time of 10 min, and 70% ethanol as a solvent. Under these conditions, the yields of the crude extracts from the roots and leaves of *Scutellaria* spp. were considerably higher than those obtained by other authors [33,43].

**Figure 1:**
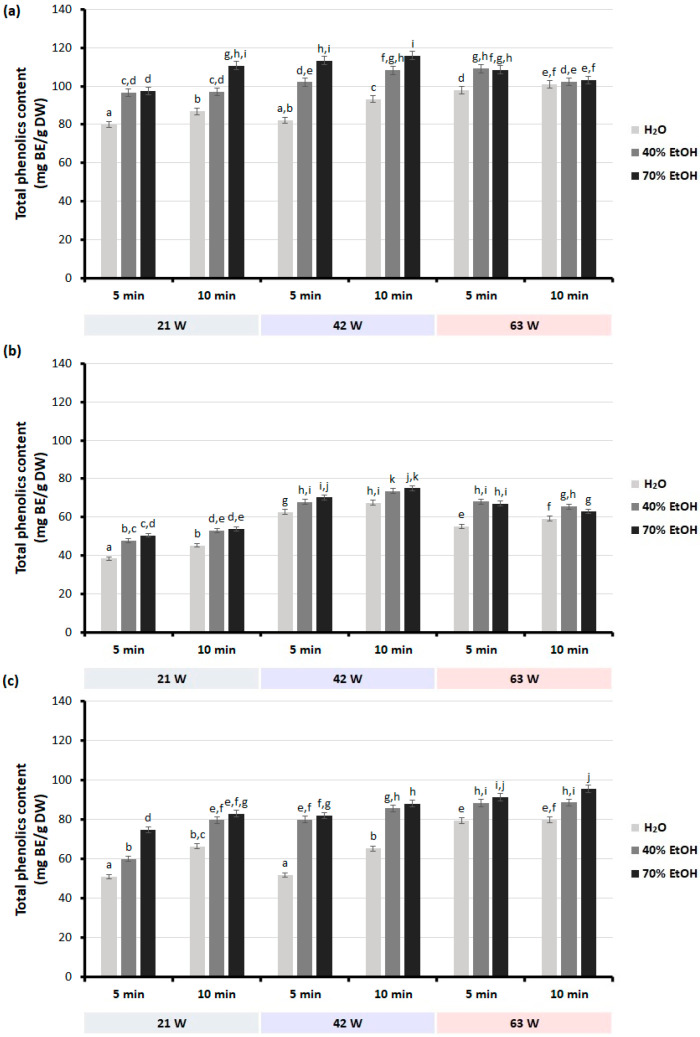
Total phenolic content in the extracts obtained from dried roots and leaves from selected *Scutellaria* species using different extraction conditions (solvent, microwave power, and extraction time): (**a**) dried roots of *S. baicalensis*; (**b**) dried leaves of *S. baicalensis*; (**c**) dried leaves of *S. lateriflora*. Results are presented as mean ± standard deviation of three replicates (*n* = 3). Bars with the same superscript (a–k) are not significantly different from each other (Tukey’s HSD test, *p* < 0.05).

The total phenolic content values of the extracts of the dried roots and leaves of *S. baicalensis* and the dried leaves of *S. lateriflora* are shown in Figure 1. The values obtained for total phenolic content are expressed as mg baicalin per g dry weight (mg BE/g DW).

The results showed that regardless of the plant material and extraction conditions (microwave power and extraction time), the use of water as a solvent resulted in extracts with the lowest total content of phenolic compounds compared to ethanolic extracts. The extraction of phenolic compounds from the aerial and underground parts of selected *Scutellaria* plants was facilitated by the use of 40 and 70% ethanol as an extractant. The highest amount of phenolics was extracted from both the roots and leaves of *S. baicalensis* and *S. lateriflora* using 70% ethanol. Higher concentrations of ethanol were not used in this study because our previous experiments [44] have shown that the use of higher concentrations of ethanol (above 70%) results in lower extraction yields and lower total phenolic content. This phenomenon may be due to the denaturation of plant tissue proteins, which blocks the release of bioactive compounds into the solution [39]. Other authors compared the efficiency of *S. baicalensis* root and *S. lateriflora* aerial part extraction with various methods using water or ethanol as solvents [33,46]. Zhou et al. (2016) [33] showed that the most effective method for the extraction of phenolic compounds from *S. baicalensis* roots was MAE using water as a solvent. They also revealed that ultrasonic-assisted extraction (UAE) with 70% ethanol produced higher yields of phenolic compounds than UAE with water [33]. Similarly, Lohan et al. (2013) showed that ethanolic extracts of *S. lateriflora* contain more phenolic compounds than water ones [46].

The total phenolic content of the extracts of the different *Scutellaria* species was also significantly affected by the microwave power and extraction time. The total phenolic content of almost all extracts obtained from selected *Scutellaria* plants was significantly increased (*p* < 0.05) upon increasing the microwave power from 21 to 63 W. The exceptions were the ethanolic extracts of the dried roots of *S. baicalensis* and all extracts of the dried leaves of the same species. Increasing the microwave power to 63 W decreased the total phenolic content of these extracts. The microwave power of 42 W was found to be the optimal value in this case. Higher microwave power did not increase the content of phenolic compounds in ethanolic extracts but increased the total phenolic content in water extracts. This may indicate that some of the extracted compounds were degraded by exposure to higher microwave powers.

Regardless of the solvent and microwave power, a longer extraction time usually resulted in higher phenolic content in the extracts. The exception was the extraction of phenolic compounds from both parts of *S. baicalensis* using 40 and 70% ethanol as a solvent and the highest microwave power (63 W). In this case, a longer extraction time resulted in lower content of phenolic compounds. This may indicate that overly long exposure to microwaves leads to the degradation or transformation of the extracted compounds. However, for the dried leaves of *S. lateriflora*, increasing the extraction time and microwave power resulted in an increase in the total phenolic content. These differences in total phenolic content in all extracts analyzed can be attributed to differences in their composition.

The total phenolic content of extracts obtained from leaves of both *S. baicalensis* and *S. lateriflora* was significantly lower than that of *S. baicalensis* root extracts. The highest total phenolic content of extracts was obtained from the dried roots of *S. baicalensis* (115.93 mg BE/g DW) when 70% ethanol was used as a solvent and the microwave power and extraction time were 42 W and 10 min, respectively. The water extract obtained from the leaves of *S. baicalensis* during 5 min extraction at 21 W was characterized by the lowest total phenolic content (38.37 mg BE/g).

According to these results, the optimal extraction conditions for obtaining the highest total phenolic content from both roots and leaves of *S. baicalensis* were found to be 70% ethanol, an extraction time of 10 min, and microwave power of 42 W. Meanwhile, in the case of the leaves of *S. lateriflora*, the optimal MAE conditions were 70% ethanol, 10 min, and 63 W.

### 2.2. Antioxidant and Anti-Inflammatory Activity of the Extracts of Selected Species of Scutellaria

**Figure 2:**
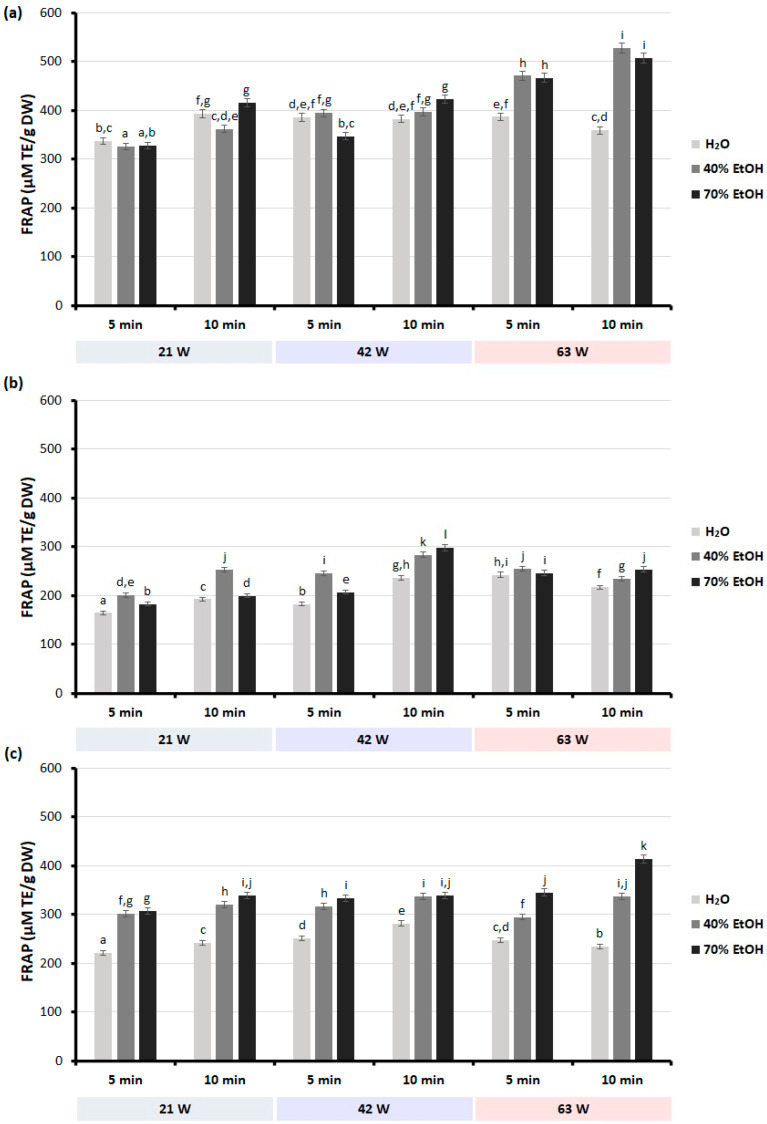
The ferric reducing ability (FRAP) of extracts obtained from dried roots and leaves from selected *Scutellaria* species: (**a**) dried roots of *S. baicalensis*; (**b**) dried leaves of *S. baicalensis*; (**c**) dried leaves of *S. lateriflora*. Results are presented as mean ± standard deviation of three replicates (*n* = 3). Bars with the same superscript (a–l) are not significantly different from each other (Tukey’s HSD test, *p* < 0.05).

**Figure 3:**
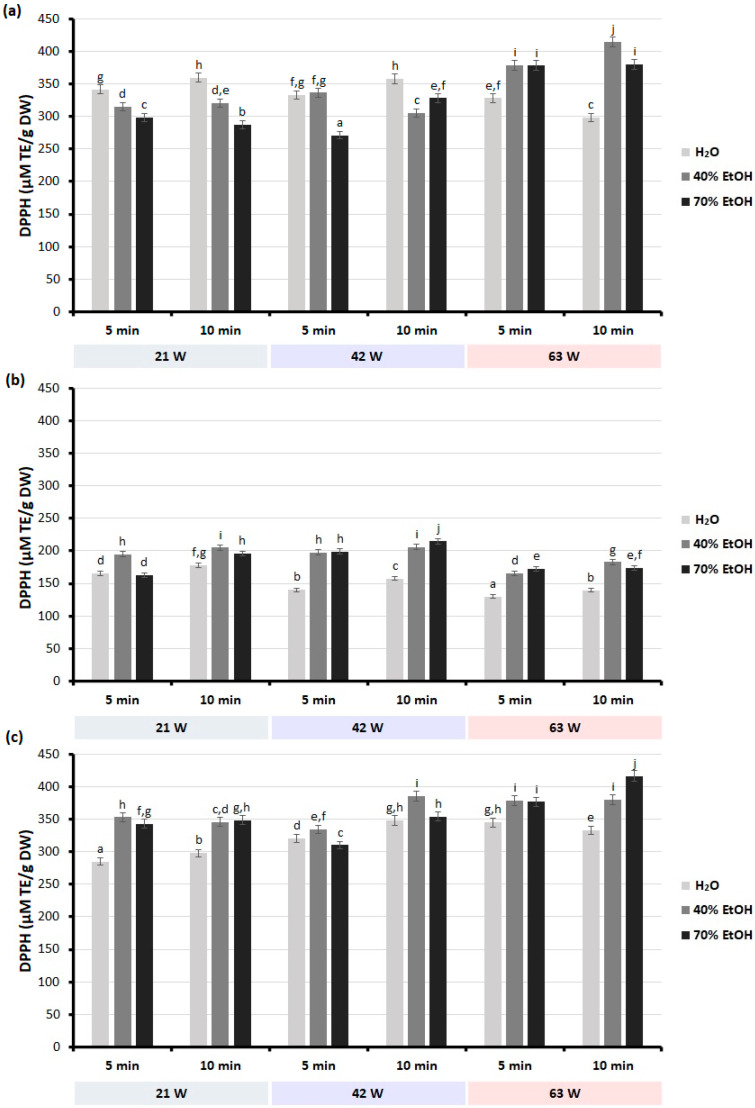
The free radical scavenging capacity (DPPH) of extracts obtained from dried roots and leaves from selected *Scutellaria* species: (**a**) dried roots of *S. baicalensis*; (**b**) dried leaves of *S. baicalensis*; (**c**) dried leaves of *S. lateriflora*. Results are presented as mean ± standard deviation of three replicates (*n* = 3). Bars with the same superscript (a–j) are not significantly different from each other (Tukey’s HSD test, *p* < 0.05).

The results showed that the conditions of the MAE process and the type of extractant have a significant effect on the antioxidant and anti-inflammatory properties of extracts from selected plant materials of the genus *Scutellaria*. Depending on the type of solvent and the extraction conditions used, the extracts obtained showed different antioxidant activity, measured using various in vitro tests (Figure 2 and Figure 3).

In general, *S. baicalensis* root extracts were characterized by higher ferric reducing activity when compared to extracts obtained from the leaves of both *Scutellaria* species (Figure 2). It was also found that the ethanolic extracts of selected *Scutellaria* plant organs had higher FRAP values than the water extracts. The ferric ion reduction ability of almost all extracts was increased upon increasing the microwave power from 21 to 63 W. Similarly, in most cases, an increase in extraction time contributed to an increase in FRAP values. The exceptions were the extracts of the dried leaves of *S. baicalensis* and water extracts of the dried leaves of *S. lateriflora*. The highest antioxidant capacity measured by the FRAP assay was noticed for 40% ethanolic extracts obtained from dried roots of *S. baicalensis* using microwave power of 63 W in 10 min (528.37 µM TE/g DW). The extract obtained from the leaves of *S. baicalensis* showed the highest activity compared to all other extracts from this material when 70% ethanol was used as a solvent and extraction was carried out for 10 min at 42 W. Similarly to the total phenolic content, the extract obtained from the leaves *S. baicalensis* after 5 min extraction at 21 W microwave power had the lowest ferric ion reduction capacity (164.35 µM TE/g DW).

The results of the present study showed also that the free radical scavenging capacity tended to increase when extending the extraction time in the case of extracts obtained from the roots and leaves of the studied *Scutellaria* species (Figure 3). Increasing the microwave power to 63 W increased the DPPH radical scavenging capacity of the extract compared to the lower microwave powers. The exception was the water extracts that were obtained from the dried leaves of *S. baicalensis*. In the case of the extracts obtained from the leaves of the two *Scutellaria* species tested, the ethanolic extracts showed a significantly greater ability to scavenge DPPH free radicals than the water extracts, irrespective of the extraction time and microwave power used. In contrast, in the case of *S. baicalensis* roots, water extracts obtained using lower microwave powers (21 and 42 W) showed higher free radical scavenging activity than ethanolic extracts. Nonetheless, of all the extracts tested, those obtained from *S. baicalensis* roots using 40% ethanol, an extraction time of 10 min, and a microwave power of 63 W had the highest ability to scavenge DPPH free radicals (414.57 µM TE/g DW). For the extract obtained from the leaves of *S. baicalensis* using water as a solvent, the shorter extraction time (5 min) and the highest microwave power (63 W) resulted in the lowest capacity for scavenging free radicals (165.87 µM TE/g DW).

The results of the DPPH radical experiments were also expressed as IC_50_ values to compare the antioxidant activity of extracts from roots and leaves of the studied *Scutellaria* species obtained under optimal MAE conditions with the results of previous studies. Under optimal conditions, the IC_50_ values of extracts from dried roots and leaves of *S. baicalensis* and dried leaves of *S. lateriflora* were found to be 38.64, 43.41, and 37.46 µg/mL, respectively (Table S1). Interestingly, the IC_50_ values obtained in the current study are significantly lower than those reported by other authors for extracts of the roots of *S. baicalensis* [33] and aerial parts of *S. lateriflora* [46]. Since a low IC_50_ value indicates strong DPPH radical scavenging activity, the results obtained confirm that the optimized extraction method yields extracts with very strong anti-free-radical properties.

In the present study, the in vitro anti-inflammatory activity of extracts obtained from the aerial and underground parts of selected *Scutellaria* spp. was also tested by evaluating their potential to inhibit the activity of lipoxygenase (LOX). Lipoxygenases are iron-containing non-heme dioxygenases. These enzymes are responsible for the formation of hydroperoxides from polyunsaturated fatty acids such as linoleic acid and arachidonic acid and for the oxidative modification of low-density lipoproteins (LDL). LOX is also involved in the progression of inflammatory processes [47,48]. During the early phase of inflammation, arachidonic acid is predominantly metabolized by 5-lipoxygenase (5-LOX), which generates a variety of mediator molecules, including highly pro-inflammatory leukotrienes such as leukotriene (LT) B4, LTC4, and LTD4 [48]. Therefore, bioactive compounds acting as LOX inhibitors are of great interest in controlling inflammatory processes [47]. The results of LOX inhibition for the extracts of the dried roots and leaves of *S. baicalensis* and the dried leaves of *S. lateriflora* are shown in Table 2. It was observed that all the extracts could inhibit the activity of this enzyme. However, the extracts obtained showed variable LOX inhibitory activity depending on the plant material, the type of solvent, and the extraction conditions used.

The extracts from the roots of *S. baicalensis* displayed stronger inhibitory activity towards LOX (IC_50_, 28.16–44.12 μg/mL) than the extracts from the leaves of the same *Scutellaria* spp. and *S. lateriflora*, with IC_50_ values in the range of 56.35–77.30 μg/mL and 39.31–61.87 μg/mL, respectively. The greatest inhibition of this enzyme was observed for the extract from the dried roots of *S. baicalensis* obtained at the microwave power of 63 W and the irradiation time of 10 min with 70% ethanol as the solvent. The lowest LOX inhibitory activity was exhibited by the water extract obtained from the leaves of the same *Scutellaria* spp. exposed to microwave power of 21 W for 5 min. LOX inhibition of the aerial and underground parts of selected *Scutellaria* spp. extracts was similar or higher than the extracts for other common plants [49]. However, it was observed that all extracts possessed significantly lower LOX inhibitory activity than pure standards (baicalin and baicalein). Nonetheless, the results of this study suggest that the extracts from both *Scutellaria* spp. plant materials, due to the presence of a high amount of bioactive compounds, were effective in inhibiting LOX activity and could reduce the production of pro-inflammatory molecules.

The leaves and roots of different *Scutellaria* spp. plants are composed of different phenolic compounds with different polarities [36,37,38,39,40,41,42]. Based on the result of the total phenolic content, antioxidant capacity, and inhibition of lipoxygenase activity of the obtained extracts, it was suggested that most of the bioactive compounds present in both *S. baicalensis* and *S. lateriflora* leaves and *S. baicalensis* roots had a moderately polar nature. Thus, the content of these compounds in the extracts increased when the ethanol concentration was increased to 70%.

### 2.3. Phenolic Profiles of the Extracts of Selected Species of Scutellaria

**Figure 4:**
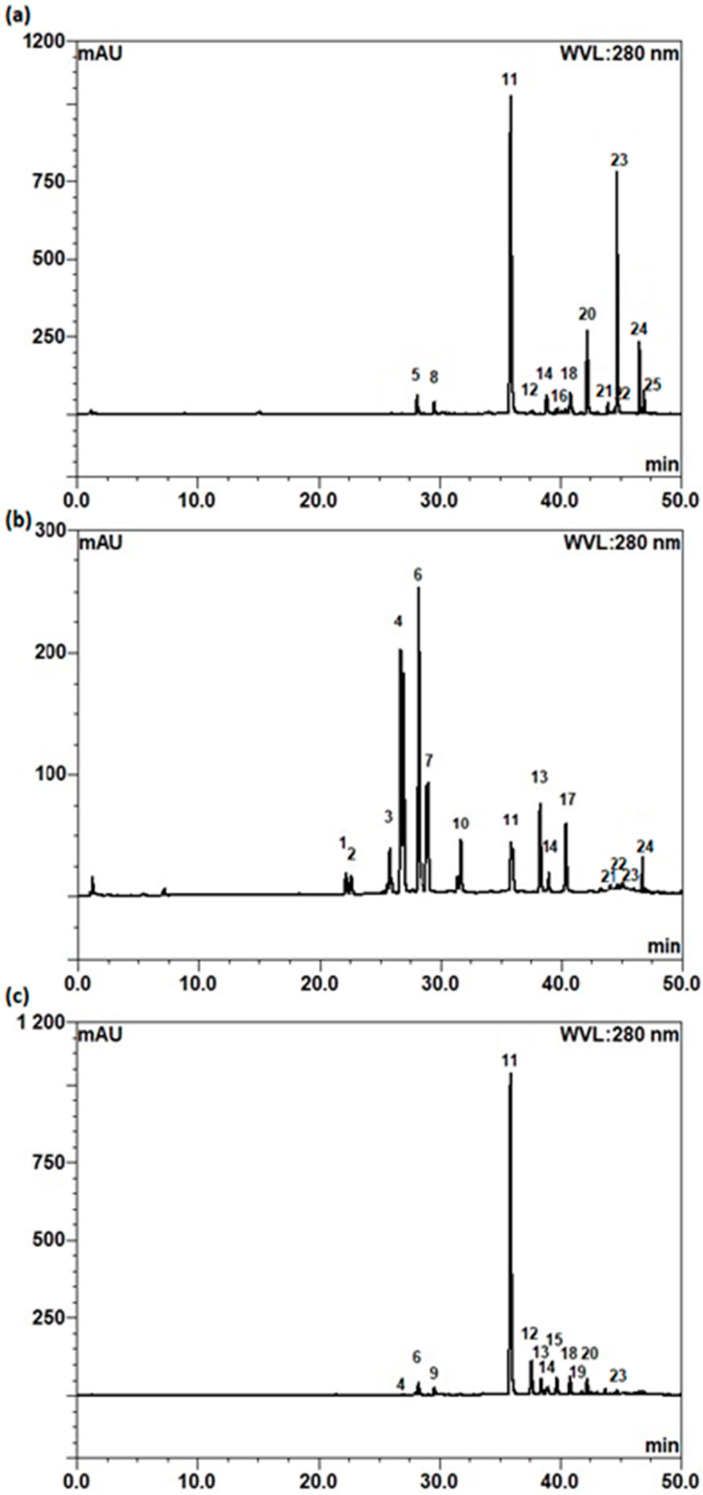
A representative chromatogram of the ethanolic extracts from selected *Scutellaria* plant materials: (**a**) dried roots of *S. baicalensis*; (**b**) dried leaves of *S. baicalensis*; (**c**) dried leaves of *S. lateriflora*. Peak identification is given in Table 3.

The characterization of the phenolic profiles of the roots and leaves of *S. baicalensis* and leaves of *S. lateriflora* extracts was performed using UHPLC–DAD/ESI–HRMS/MS. Table 3 shows the phenolic compounds tentatively identified on the basis of absorption maxima in UV–vis spectra, full-scan mass spectra, MS/MS fragmentation patterns, retention times, and chromatographic elution order, and a comparison with standard compounds and literature data [50,51,52,53,54,55]. The typical chromatograms of the extracts of selected plant materials belonging to the genus *Scutellaria* are shown in Figure 4, and the structures of the identified phenolic compounds are shown in Figure S1.

In this study, a total of 16 phenolic compounds were identified in the roots of *S. baicalensis*, a total of 15 in the leaves of *S. baicalensis*, and 15 in the leaves of *S. lateriflora*, all of which were flavonoids, mainly flavones, flavanones, and their derivatives.

The roots of *S. baicalensis* were dominated by the presence of baicalin, followed by baicalein, wogonin 7-*O*-glucuronide, wogonin, oroxylin A 7-*O*-glucuronide, norwogonin 7-*O*-glucuronide, oroxylin A, chrysin 6-*C*-arabinoside-8-*C*-glucoside, chrysin 6-*C*-glucoside-8-*C*-arabinoside, norwogonin, chrysin 7-*O*-glucuronide, hydroxyldimethoxyflavanone glucuronide, hispidulin, baicalein 6-*O*-glucuronide, oroxylin A 7-*O*-glucoside, and dihydrobaicalein 7-*O*-glucuronide.

It is interesting to note that the leaves of both *Scutellaria* species showed a different profile of phenolic compounds. The compounds that were detected in the aerial parts of *S. baicalensis* were isocarthamidin 7-*O*-glucuronide, scutellarein 7-*O*-glucuronide, carthamidin 7-*O*-glucuronide, hispidulin 7-*O*-glucuronide, baicalin, apigenin 6-*C*-glucoside-8-*C*-arabinoside, apigenin 7-*O*-glucuronide, 5,6,7,3′,4′-pentahydroxyflavanon 7-*O*-glucoronide isomers, norwogonin 7-*O*-glucuronide, baicalein, hispidulin, norwogonin, and wogonin. In contrast, the leaves of *S. lateriflora* were mainly characterized by the presence of baicalin, followed by glucuronides of dihydrobaicalein, oroxylin A, wogonin, hydroxyldimethoxyflavanone, hispidulin, norwogonin, isoscutellarein, and scutellarein. In addition, baicalein, chrysin 6-*C*-arabinoside-8-*C*-glucoside, and glucuronides of apigenin, isocarthamidin, and chrysin have been identified in the leaves of this species. These compounds were previously reported in the roots and leaves of different *Scutellaria* spp. plants [50,51,52,53,54].

Both qualitative and quantitative differences in the composition of phenolic compounds were observed among the different species of *Scutellaria* and the different MAE parameters (Table 4, Table 5 and Table 6). The total amount of identified phenolic compounds, calculated as the sum of individual compounds, in the obtained extracts ranged from 1.03 to 102.75 mg/g DW.

The ethanolic extracts obtained from the aerial and underground parts of the selected *Scutellaria* species showed significantly (*p* < 0.05) higher content of phenolic compounds than those obtained with water as the solvent, regardless of the extraction conditions used. In addition, the total amount of phenolic compounds in the extracts of all plant materials tested increased when the ethanol concentration was increased from 40 to 70%. It was also confirmed by other authors that 70% ethanol is the best solvent for the extraction of the flavonoids from the different species of the genus *Scutellaria* [40]. However, Zu et al. (2016) [33] demonstrated that microwave-assisted extraction using water at 300 W at 100 °C for 2 h was the most efficient method for the extraction of phenolic compounds from *S. baicalensis* roots. Bergeron et al. (2005) [41] showed that extraction with either water at 85 °C for 30 min or with 70% ethanol for 24 h at room temperature yields the highest amounts of flavonoids present in the leaves of *S. lateriflora*. Besides the fact that the solubility of the majority of substances usually increases with temperature, it is also believed that a high temperature reduces the polarity of water and increases its ability to dissolve non-polar compounds such as baicalin, baicalein, scutellarin, lateriflorin, dihydrobaicalin, and oroxylin A 7-*O*-glucuronide [41]. Due to increased solvent diffusion into the plant matrix and the increased solubility and desorption of phenolic compounds from the matrix, higher temperatures result in higher extraction yields. However, when higher temperatures are used, the degradation of heat-sensitive compounds may occur [55]. In the present study, the extraction temperature did not exceed 60 °C. Therefore, water was the least effective solvent for the flavonoids present in *Scutellaria* roots and leaves.

The concentration of phenolic compounds in the extracts of the different *Scutellaria* species was also considerably affected by the extraction time and microwave power (Table 4, Table 5 and Table 6). It was also observed that the extraction efficiency of phenolic compounds increased as the extraction time increased from 5 to 10 min. The total phenolic compound content of almost all extracts obtained was also increased upon increasing the microwave power from 21 to 63 W.

Furthermore, it was found that ethanolic extracts obtained from the roots of *S. baicalensis* had significantly higher content of total phenolic compounds (81.15–102.75 mg/g DW) than those obtained from the leaves of both species (30.75–51.95 mg/g DW), regardless of the ethanol concentration and extraction conditions used. The highest total quantity of the selected individual phenolic compounds was observed for the extract obtained from the roots of *S. baicalensis* with 70% ethanol for 10 min at the microwave power of 63 W, while the lowest concentration of phenolic compounds was found in the water extract obtained from the leaves of *S. lateriflora* during 5 min extraction at 21 W.

The main phenolic compound in ethanolic extracts from the roots of *S. baicalensis* was baicalin (42.97–52.66 mg/g DW), followed by baicalein (15.09–17.64 mg/g DW), wogonoside (7.19–12.07 mg/g DW), wogonin (4.02–4.59 mg/g DW), oroxyloside (2.06–3.61 mg/g DW), norwogonoside (1.43–3.26 mg/g DW), chrysin 6-*C*-arabinoside-8-*C*-glucoside (1.87–2.12 mg/g DW), norwogonin (0.96–1.53 mg/g DW), chrysin 6-*C*-glucoside-8-*C*-arabinoside (0.87–1.48 mg/g DW), and oroxylin A (1.35–1.71 mg/g DW). The levels of other compounds that were detected in these extracts varied from 0.06 to 0.91 mg/g of DW.

The ethanolic extracts of the leaves of *S. baicalensis* were dominated by isocarthamidin 7-O-glucuronide (10.74–18.41 mg/g DW), followed by scutellarin (6.34–9.95 mg/g DW), hispiduloside (1.98–3.47 mg/g DW), baicalin (1.82–3.32 mg/g DW), carthamidin 7-*O*-glucuronide (1.07–3.33 mg/g DW), chrysin 7-*O*-glucuronide (1.55–2.54 mg/g DW), apigenin 7-*O*-glucuronide (1.11–1.84 mg/g DW), and apigenin 6-*C*-glucoside-8-*C*-arabinoside (1.10–1.76 mg/g DW). The levels of other phenolic compounds were much lower (0.06–0.93 mg/g DW). The total content of the investigated phenolic compounds in the ethanolic extracts of the leaves of *S. baicalensis* varied from 30.75 to 46.48 mg/g DW.

Baicalin (31.14–36.33 mg/g DW) was detected in the highest concentration in the ethanolic extracts of the leaves of *S. lateriflora*, followed by dihydrobaicalin (3.30–4.38 mg/g DW). The levels of oroxyloside, hydroxyldimethoxyflavanone glucuronide, wogonoside hispiduloside, baicalein, and scutellarin in the ethanolic extracts of these leaves depended on the ethanol concentration and extraction conditions and fluctuated within the range of 1.69–2.04, 1.57–1.93, 1.53–1.90, 1.53–1.84, 0.48–1.61, and 0.96–1.16 mg/g DW, respectively. The other phenolic compounds were found in much lower concentrations in *S. lateriflora* ethanolic extracts (0.01–0.76 mg/g DW). The total content of the investigated phenolic compounds in the ethanolic extracts of these leaves ranged from 44.60 to 51.95 mg/g DW. The differences in the profiles and levels of phenolic compounds between the different morphological parts of the *Scutellaria* plants were due to the features of the different species [50,51,52,53,54].

Interestingly, the water extracts from the leaves of *S. baicalensis* were characterized by a significantly higher total concentration of phenolic compounds (5.94–7.91 mg/g DW), compared to the water extracts from the other aerial and underground parts of the studied *Scutellaria* species (1.03–4.36 mg/g DW). The leaves of *S. baicalensis* differed from the roots of the same species and the leaves of *S. lateriflora* by the presence of high amounts of tetrahydroxyflavanone and tetrahydroxyflavone glucuronides, mainly isocarthamidin 7-*O*-glucuronide and scutellarin, structurally related to (S)-naringenin and apigenin, respectively. These compounds are more hydrophilic than baicalin, baicalein, and dihydrobaicalin, the major phenolic compounds present in *S. baicalensis* roots and *S. lateriflora* leaves, and can be efficiently extracted even when water is used as a solvent. Thus, the two predominant compounds in water extracts of *S. baicalensis* were isocarthamidin 7-*O*-glucuronide (1.39–1.95 mg/g DW) and scutellarin (1.39–1.91 mg/g DW), and these were followed by apigenin 6-*C*-glucoside-8-*C*-arabinoside (0.71–1.37 mg/g DW), baicalin (0.55–0.91 mg/g DW), hispiduloside (0.23–0.63 mg/g DW), apigenin 7-*O*-glucuronide (0.42–0.56 mg/g DW), carthamidin 7-*O*-glucuronide (0.29–0.44 mg/g DW), wogonin (0.10–0.26 mg/g DW), and 5,6,7,3′,4′-pentahydroxyflavanon 7-*O*-glucoronide isomers (0.09–0.26 mg/g DW). The other detected phenolic compounds were present in trace amounts.

In contrast, much fewer phenolic compounds were detected in the water extracts of the roots of *S. baicalensis* and leaves of *S. lateriflora* (Table 4, Table 5 and Table 6). The water extract of *S. baicalensis* roots was dominated by chrysin 6-*C*-arabinoside-8-*C*-glucoside (0.99–1.75 mg/g DW) and chrysin 6-*C*-glucoside-8-*C*-arabinoside (0.66–1.18 mg/g DW). Interestingly, the content of these two compounds was only slightly lower compared to their content in ethanolic extracts. Meanwhile, the content of baicalin (0.46–1.22 mg/g DW) and baicalein (0.04–0.13 mg/g DW) in water extracts of this raw material was more than 40-fold and 135-fold lower, respectively, compared to ethanolic extracts. The other compounds detected were present only in trace amounts. These observations agree with the findings reported by Zhou et al. (2016) [33]. They also showed that when water was used as a solvent in the ultrasound-assisted extraction of *S. baicalensis* roots, the obtained extracts had significantly lower amounts of baicalin, baicalein, wogonoside, and wogonin than the UAE extracts obtained using 70% ethanol. However, the amount of water-soluble components such as chrysin 6-*C*-arabinoside-8-*C*-glucoside and chrysin 6-*C*-glucoside-8-*C*-arabinoside was only slightly lower [33].

Similar trends were observed for the water and ethanolic extracts of the leaves of *S. lateriflora*. Almost all phenolic compounds detected in water extracts of this plant material, especially baicalin, dihydrobaicalin, and baicalein, were found in lower concentrations than in ethanolic extracts. For the water extract of *S. lateriflora* leaves, the lowest recovery compared to the ethanolic extract was found for dihydrobaicalin. This compound was the second most abundant phenolic in the ethanolic extracts of this material, similar to the findings reported by Bergeron et al. (2005) [41]. In the present study, only trace amounts of dihydrobaicalin (0.02–0.03 mg/g DW) were found in the water extract, and only when the highest microwave power (63 W) was used during extraction. Other authors have also shown that water extracts of *S. lateriflora* leaves contain less dihydrobaicalin than ethanolic extracts [41].

It is not surprising that the total concentration of phenolic compounds determined by the UHPLC–DAD method differs from that obtained by the Folin–Ciocalteu (F-C) assay (Figure 1 and Table 4, Table 5 and Table 6). The F-C method is often used to determine the total phenolic content in plant extracts. However, this assay is not specific to phenolic compounds only. Other non-phenolic-reducing agents such as ascorbic acid, sugars, amino acids, peptides, organic acids, aromatic amines, and Maillard reaction products may react with the F-C reagent and interfere with the determination results. The F-C test can be used to determine not only phenolic compounds but also other substances with reducing capacity [44,56]. Therefore, the results of the phenolic compounds determined by the F-C test and the UHPLC–DAD method may differ significantly. These differences may be mainly due to differences in the content of individual phenolic compounds in the obtained extracts, but they may also result from the transfer of other compounds present in the tested plant material to the extract, such as polysaccharides and their degradation products formed during microwave extraction [57].

In conclusion, the results obtained show that extraction with 70% ethanol at 10 min extraction time and microwave power of 63 W are the optimal MAE processing conditions for obtaining the highest content of most of the compounds analyzed, especially baicalin, baiclalein, wogonoside, wogonin, and isocarthamidin 7-*O*-glucuronide, from the roots and leaves of *S. baicalensis* and leaves of *S. lateriflora*.

The results of this study confirm the strong antioxidant properties of *S. baicalensis* root and *S. lateriflora* leaf extracts obtained by microwave extraction, suggesting their beneficial potential in the prevention of diseases associated with oxidative stress, such as cardiovascular and inflammatory diseases. The flavonoids contained in these extracts, especially baicalein and its glucuronides, may be responsible for their high antioxidant activity.

In vitro studies confirmed that baicalein can scavenge hydrogen peroxide, superoxide, and even hydroxyl radicals. These findings were confirmed in an ischemia–reperfusion model where the treatment of cardiomyocytes with *S. baicalensis* root extract reduced cell death from almost 50 to 23% [58]. The protective effect has also been confirmed in experiments using extracts from two other species of *Scutellaria*. Extracts from *S. altissima* and *S. alpina* have been proven to protect against oxidative damage to human plasma proteins and lipids [59], as well as blood platelets and lymphocytes [60]. Previous studies have shown that the major bioactive compounds found in *Scutellaria* spp., including baicalein, baicalin, wogonin, wogonoside, and oroxylin A, can inhibit the production of several pro-inflammatory cytokines and chemokines and pro-inflammatory mediators, while promoting the production of certain anti-inflammatory cytokines [25,26,61].

## 3. Materials and Methods

### 3.1. Chemical Reagents and Materials

Baicalein, baicalin, acetonitrile of HPLC grade (≥99.9%), 6-hydroxy-2,5,7,8-tetramethylchroman-2-carboxylic acid (Trolox), 2,2-diphenyl-1-picrylhydrazyl (DPPH), 2,4,6-tri(2-pyridyl)-s-triazine (TPTZ), ferric chloride hexahydrate, sodium acetate, lipoxidase from *Glycine max* (type V), and linoleic acid were all obtained from Sigma-Aldrich (St. Louis, MO, USA). Water was purified by a Milli-Q water purification system (Millipore Corp., Bedford, MA, USA). All other chemicals were of analytical grade.

### 3.2. Plant Materials

The dried roots of *S. baicalensis* were purchased from Nanga (Blękwit, Poland) and dried leaves of S. *baicalensis* and *S. lateriflora* were obtained from Plantago (Złotów, Poland). All the samples were dried plant materials. Before the extraction, the plant materials were ground to powder (<0.50 mm) using a laboratory knife mill (GRINDOMIX GM 200, Retsch, Haan, Germany).

### 3.3. Microwave-Assisted Extraction (MAE)

The leaf and root powders were extracted in Teflon vessels using an RM-800 microwave reactor (Plazmatronika, Poland, Wroclaw) at a temperature not exceeding 60 °C, with continuous stirring using a built-in magnetic stirrer. The temperature during extraction was automatically and continuously measured and controlled by a Reflex fiber-optic temperature meter (Neoptix, Canada, Québec). MAE was performed using three different solvents (water, EtOH/H_2_O 40:60 *v*/*v*, and EtOH/H_2_O 70:30 *v*/*v*). The MAE parameters were the microwave power (21, 42, and 63 W) and extraction time (5 and 10 min). The ratio of dry plant material to solvent used was 1:20 (*w/v*). Each extraction experiment was performed in triplicate. We selected the extraction parameters based on literature data [41] and our previous experience with other plants [44], as data on the use of microwaves to extract phenolic compounds from *Scutellaria* plants are still limited. After MAE, the extract was centrifuged at 1050× *g* for 10 min at 20 °C in the centrifuge type MPW-350 (MPW MED, Instruments, Warsaw, Poland). The supernatants were filtered through Whatman No. 4 filter paper and stored at −80 °C until further use. To determine the extraction yield, 10 mL of each supernatant was transferred to a pre-weighed 40 mL glass vial. Supernatants were evaporated to dryness at 40 °C.

The extraction yield was calculated using the following equation:

Extraction yield (mg/g) = (X_1_/X_2_) (1)

where X_1_ is the weight (mg) of the dried extract and X_2_ is the weight (g) of the plant material subjected to the extraction process.

### 3.4. Identification and Quantification of Polyphenolic Compounds by UHPLC–DAD/ESI–HRMS/MS

UHPLC–DAD and UHPLC–ESI–HRMS/MS analyses of polyphenolic compounds in *Scutellaria* plant extracts were carried out using a UHPLC+ Dionex UltiMate 3000 liquid chromatographic system (Thermo Fisher Scientific Inc., Waltham, MA, USA) equipped with a diode array detector and a Transcend™ TLX-2 multiplexed LC system coupled to a Q-Exactive hybrid quadrupole-orbitrap mass spectrometer with a heated electrospray ionization (HESI–II) source (Thermo Scientific, Hudson, NH, USA), respectively. Prior to analysis, the extracts were filtered through 0.20-μm nylon syringe filters. Chromatographic separation was performed on the Accucore™ C18 column (2.1 × 150 mm, 2.6 μm particle size; Thermo Scientific, PA, USA) in the gradient elution mode. The column was maintained at 30 °C and the flow rate was 0.35 mL/min. Eluent A was 0.1% formic acid in water and eluent B was acetonitrile. The gradient elution program was as follows: 0–8 min, 1–5% B; 8–15 min, 5–8% B; 15–20 min, 8–10% B; 20–25 min, 10–15% B; 25–35 min, 15–20% B; 35–40 min, 20–25% B; 40–50 min, 25–90% B; 50–53 min, 90% B; 53–58 min, 90–1% B; 58–65 min, 1% B. The injection volume was set to 10 μL. The chromatograms were recorded at 280 nm. The mass spectrometer was operated in negative ionization mode with full MS-SIM and a subsequent parallel reaction monitoring (PRM) mode. Full-MS-SIM and PRM spectra were obtained by scanning *m*/*z* from 100 to 1500. The mass spectrometer operation conditions were as follows: capillary temperature, 275 °C; heater gas temperature, 320 °C; electrospray capillary voltage, 4.5 kV; sheath and auxiliary gas, nitrogen; sheath gas flow, 35 arbitrary units; auxiliary gas flow, 15 arbitrary units; normalized collision energy (NCE), 20% [35]. The identification of polyphenolic compounds was performed by matching their retention times, spectra characteristics, full-scan mass spectra in negative ionization mode, and MS/MS fragmentation patterns with those of pure standards analyzed under identical conditions and literature data [50,51,52,53,54]. Quantification of individual polyphenolic compounds was carried out using the external standard method. All measurements were conducted in triplicate and results were expressed as mg per g DW (mg/g DW).

### 3.5. Determination of Total Phenolic Content

The total phenolic content of the extracts of selected *Scutellaria* plant materials was determined using the Folin–Ciocalteu method, as described by Oracz and Żyżelewicz (2019) [62]. The total polyphenolic content was calculated from the calibration curve prepared with the use of baicalin as the standard. Results of the total phenolic content were expressed as mg baicalin equivalents (BE) per gram of DW (mg BE/g DW).

### 3.6. Evaluation of the Antioxidant Activity

Two in vitro assays, namely free radical-scavenging capacity (DPPH) and ferric ion reducing antioxidant power (FRAP), were used to evaluate the antioxidant potential of the extracts of selected *Scutellaria* plant materials, as described by Oracz and Żyżelewicz (2019) [62]. The results of the DPPH and FRAP assays were expressed as µM Trolox equivalents per gram of DW (μM TE/g DW).

### 3.7. Evaluation of the Anti-Inflammatory Activity

In vitro inhibition of LOX activity was determined spectrophotometrically according to Michel et al. (2017) [63], with some minor modifications. Briefly, 50 μL of tested extract (50 to 500 μg/mL) was mixed with 950 μL of sodium borate buffer (pH = 9.0), 1 mL of LOX solution (300 U/mL), and 1 mL of linoleic acid solution (138 μM). The increase in absorbance of the reaction mixture was measured for each sample on a UV-1800 spectrophotometer (Shimadzu, Tokyo, Japan) at 234 nm over a period of 15 min. Baicalein and baicalin were used as reference standards, whereas sodium borate buffer solution was used as the control. The activity in the absence of the extract or standard was considered to be 100% of LOX activity.

The anti-inflammatory effect was evaluated by calculating the percentage inhibition of hydroperoxide production from the absorbance values at 234 nm after 10 min of incubation. The results of LOX inhibitory activity were expressed as the IC_50_ value (μg/mL); a lower IC_50_ value represents stronger anti-inflammatory activity.

### 3.8. Statistical Analysis

The results of three independent experiments were expressed as the mean ± standard deviation (SD). The Statistica 13.0 software (StatSoft, Inc., Tulsa, OK, USA) was used for the statistical data analyses. The significant differences were estimated by one-way analysis of variance (ANOVA) followed by Tukey’s Honest Significant Difference (HSD) test. Differences were considered to be significant when *p*-values were less than 0.05 (*p* < 0.05).

## 4. Conclusions

This study reported the manipulation of the extraction conditions of the dried roots and leaves of *S. baicalensis* and dried leaves of *S. lateriflora* using the MAE method in order to obtain extracts with higher levels of phenolic compounds. The results of the research allowed the optimization of the microwave extraction process, including the choice of solvent, time, and microwave power, to maximize the total and individual phenolic compounds and the antioxidant and anti-inflammatory activity of extracts from the aerial and underground parts of selected *Scutellaria* plants. The optimal MAE processing conditions for obtaining the extracts from the roots and leaves of *S. baicalensis* and leaves of *S. lateriflora*, with the highest total and individual content of most phenolic compounds, especially baicalin, baicalein, wogonoside, wogonin, and isocarthamidin 7-*O*-glucuronide, and enhanced antioxidant and anti-inflammatory potential, were 70% ethanol as a solvent, an extraction time of 10 min, and microwave power of 63 W. It was found that under these conditions, MAE was a more efficient process than heat reflux and UAE for the extraction of bioactive compounds from the roots and leaves of selected *Scutellaria* species. In addition, the extracts obtained by the optimized extraction procedure showed significant antioxidant and anti-inflammatory activity and could be used as a natural source of bioactive compounds in the production of nutraceuticals and functional foods. The results of this study may be useful for the development of industrial MAE processes with improved extraction efficiency for selected bioactive compounds from different organs of *S. baicalensis* and *S. lateriflora*.

## Figures and Tables

**Table 1:** The yield of crude extracts obtained from dried roots and leaves of *S. baicalensis* and dried leaves of *S. lateriflora*.

Extraction Conditions			Yield of Crude Extract (mg/g)		
Solvent	Microwave Power	Time	SBR	SBL	SLL
H_2_O	21 W	5 min	349.16 ± 0.85 ^a^	249.36 ± 0.76 ^a^	279.77 ± 0.68 ^a^
		10 min	351.82 ± 0.79 ^a^	324.22 ± 0.82 ^g^	323.92 ± 0.71 ^c^
	42 W	5 min	436.47 ± 0.82 ^f^	321.45 ± 0.75 ^f,g^	341.15 ± 0.73 ^g,h^
		10 min	444.71 ± 0.76 ^g^	348.19 ± 0.72 ^k^	343.91 ± 0.75 ^h,i^
	63 W	5 min	443.03 ± 0.81 ^g^	334.65 ± 0.74 ^i^	338.30 ± 0.81 ^f,g^
		10 min	453.44 ± 0.73 ^i^	355.57 ± 0.67 ^l^	359.95 ± 0.83 ^j^
40% EtOH	21 W	5 min	381.21 ± 0.77 ^b^	298.66 ± 0.73 ^b^	309.63 ± 0.73 ^b^
		10 min	403.08 ± 0.81 ^d^	311.50 ± 0.72 ^d^	327.78 ± 0.76 ^d^
	42 W	5 min	417.10 ± 0.79 ^e^	304.56 ± 0.76 ^c^	345.43 ± 0.75 ^i^
		10 min	467.60 ± 0.87 ^k^	320.70 ± 0.80 ^e,f^	335.44 ± 0.72 ^e,f^
	63 W	5 min	463.03 ± 0.85 ^j^	318.08 ± 0.76 ^e^	332.39 ± 0.81 ^e^
		10 min	473.44 ± 0.82 ^l,m^	331.31 ± 0.72 ^h^	344.48 ± 0.76 ^i^
70% EtOH	21 W	5 min	388.48 ± 0.75 ^c^	329.39 ± 0.81 ^h^	341.31 ± 0.75 ^g,h^
		10 min	449.14 ± 0.79 ^h^	347.96 ± 0.82 ^j,k^	360.12 ± 0.72 ^j^
	42 W	5 min	461.08 ± 0.81 ^j^	345.09 ± 0.69 ^j^	366.11 ± 0.81 ^k^
		10 min	472.12 ± 0.83 ^l^	359.91 ± 0.76 ^m^	378.73 ± 0.78 ^l^
	63 W	5 min	477.10 ± 0.78 ^m^	349.79 ± 0.73 ^k^	367.73 ± 0.74 ^k^
		10 min	488.03 ± 0.76 ^n^	373.47 ± 0.81 ^n^	386.05 ± 0.75 ^m^

Results are presented as the mean ±SD from triplicate determinations, and values followed by different superscript letters (a–n) in the same column are significantly different (Tukey’s HSD test, *p* < 0.05); SBR—dried roots of *S. baicalensis*; SBL—dried leaves of *S. baicalensis*; SLL—dried leaves of *S. lateriflora*.

**Table 2:** The lipoxygenase inhibition by extracts obtained from dried roots and leaves of *S. baicalensis* and dried leaves of *S. lateriflora*.

Plant Material	Extraction Conditions			LOX Inhibition (IC_50_, µg/mL)
	Solvent	Microwave Power	Time	
Root of *S. baicalensis*	H_2_O	21 W	5 min	40.78 ± 0.12 ^k^
			10 min	29.45 ± 0.15 ^j,k^
		42 W	5 min	33.47 ± 0.13 ^f^
			10 min	36.86 ± 0.10 ^h^
		63 W	5 min	39.72 ± 0.11 ^j^
			10 min	44.12 ± 0.09 ^l^
	40% EtOH	21 W	5 min	37.29 ± 0.15 ^h^
			10 min	32.25 ± 0.14 ^e^
		42 W	5 min	38.93 ± 0.13 ^i^
			10 min	33.90 ± 0.11 ^f^
		63 W	5 min	38.60 ± 0.11 ^i^
			10 min	30.80 ± 0.13 ^c,d^
	70% EtOH	21 W	5 min	35.77 ± 0.12 ^g^
			10 min	30.59 ± 0.13 ^c,d^
		42 W	5 min	31.08 ± 0.10 ^d^
			10 min	29.40 ± 0.11 ^b^
		63 W	5 min	30.24 ± 0.14 ^c^
			10 min	28.16 ± 0.13 ^a^
Leaves of *S. baicalensis*	H_2_O	21 W	5 min	71.60 ± 0.15 ^k^
			10 min	68.55 ± 0.10 ^i^
		42 W	5 min	63.33 ± 0.11 ^f^
			10 min	67.77 ± 0.12 ^h^
		63 W	5 min	71.53 ± 0.14 ^k^
			10 min	77.30 ± 0.15 ^m^
	40% EtOH	21 W	5 min	68.33 ± 0.12 ^h,i^
			10 min	61.72 ± 0.13 ^e^
		42 W	5 min	70.49 ± 0.16 ^j^
			10 min	63.88 ± 0.11 ^f^
		63 W	5 min	70.05 ± 0.12 ^j^
			10 min	59.82 ± 0.10 ^c,d^
	70% EtOH	21 W	5 min	66.33 ± 0.14 ^g^
			10 min	59.54 ± 0.13 ^c,d^
		42 W	5 min	60.18 ± 0.12 ^d^
			10 min	57.98 ± 0.11 ^b^
		63 W	5 min	59.07 ± 0.13 ^c^
			10 min	56.35 ± 0.12 ^a^
Leaves of *S. lateriflora*	H_2_O	21 W	5 min	61.87 ± 0.14 ^k^
			10 min	54.58 ± 0.12 ^f^
		42 W	5 min	56.52 ± 0.13 ^i^
			10 min	49.70 ± 0.11 ^c^
		63 W	5 min	55.81 ± 0.10 ^g,h^
			10 min	49.21 ± 0.13 ^c^
	40% EtOH	21 W	5 min	61.80 ± 0.14 ^k^
			10 min	54.53 ± 0.11 ^f^
		42 W	5 min	56.45 ± 0.12 ^h,i^
			10 min	49.66 ± 0.15 ^c^
		63 W	5 min	55.74 ± 0.10 ^g^
			10 min	49.42 ± 0.15 ^c^
	70% EtOH	21 W	5 min	58.05 ± 0.13 ^j^
			10 min	52.03 ± 0.12 ^d^
		42 W	5 min	52.95 ± 0.13 ^e^
			10 min	42.32 ± 0.14 ^b^
		63 W	5 min	42.27 ± 0.12 ^b^
			10 min	39.31 ± 0.10 ^a^
Baicalein				1.35 ± 0.05
Baicalin				2.30 ± 0.07

Results are presented as the mean ±SD from triplicate determinations, and values followed by different superscript letters (a–m) in the same column are significantly different (Tukey’s HSD test, *p* < 0.05); SBR—dried roots of *S. baicalensis*; SBL—dried leaves of *S. baicalensis*; SLL—dried leaves of *S. lateriflora*.

**Table 3:** The phenolic compounds tentatively identified by UHPLC–DAD/ESI–HRMS/MS method in dried roots and leaves of *S. baicalensis* and dried leaves of *S. lateriflora* extracts.

Peak No.	R_t_ (min)	λ_max_ (nm)	[M−H]^−^ (*m/z*)	Fragment Ions (*m/z*)	Compound Identified	Abbreviation	References	SBR	SBL	SLL
1	22.32	285.8, 361.7	479.0858	347.0673, 285.0969	5,6,7,3′,4′-Pentahydroxyflavanon 7-*O*-glucoronide	Phf-GluA	[50]	-	+	-
2	22.73	285.3, 361.1	479.0858	347.0673, 285.0969	5,6,7,3′,4′-Pentahydroxyflavanon 7-*O*-glucoronide isomer	Phf-GluA iso	[50]	-	+	-
3	25.97	268.9, 335.2	563.1438	479.0851, 285.0971	Apigenin 6-*C*-glucoside-8-*C*-arabinoside	Ap-Glu-Ara	[50,51]	-	+	-
4	26.85	286.0, 361.2	463.0909	287.0575, 269.0468, 193.0356, 175.0245	Isocarthamidin 7-*O*-glucuronide	IsoCart-GluA	[50,51]	-	+	+
5	28.13	272.7, 314.3	547.1489	217.1085, 145.0500	Chrysin 6-*C*-arabinoside-8-*C*-glucoside	Chry-Ara-Glu	[50,51]	+	-	+
6	28.30	282.4, 333.8	461.0750	285.0417, 175.0245, 113.0234	Scutellarein 7-*O*-glucuronide (scutellarin)	Scu-GluA	[50,51]	-	+	+
7	29.00	287.3, 362.9	463.0910	287.0575, 269.0468, 193.0356, 175.0245	Carthamidin 7-*O*-glucuronide	Cat-GluA	[50,51]	-	+	-
8	29.58	272.7, 314.4	547.1491	217.1085, 145.0500	Chrysin 6-*C*-glucoside-8-*C*-arabinoside	Chry-Glu-Ara	[50,51]	+	-	-
9	29.64	273.4, 333.8	461.0754	285.0419, 175.0245, 113.0234	Isoscutellarein 7-*O*-glucuronide	Isoscu-GluA	[50]	-	-	+
10	31.79	266.7, 336.7	445.0799	269.0452	Apigenin 7-*O*-glucuronide	Ap-GluA	[50,51,52,53]	-	+	+
11	35.93	377.1, 316.1	445.0794	269.0468, 175.0245, 113.0234	Baicalein 7-O-glucuronide (baicalin)	Baic-7-GluA	[50,51]	+	+	+
12	37.68	237.4, 287.8, 361.0	447.0956	271.0625, 217.1085, 145.0500	Dihydrobaicalein 7-O-glucuronide (dihydrobaicalin)	Dihbaic-GluA	[54]	+	-	+
13	38.28	274.1, 332.7	475.0905	299.0556, 217.1085, 145.0500	Hispidulin 7-*O*-glucuronide (hispiduloside)	Hisp-GluA	[53,54]	-	+	+
14	38.94	279.2, 357.5	445.0802	269.0455	Norwogonin 7-*O*-glucuronide (norwogonoside)	Norw-GluA	[51]	+	+	+
15	39.79	284.2, 357.7	475.0909	299.0568, 217.1085	Hydroxyldimethoxyflavanone glucuronide	Hdimf-GluA	[52]	+	-	+
16	40.15	272.7, 310.8	445.0802	269.0455, 175.02,	Oroxylin A 7-*O*-glucoside	Orx-Glu	[52]	+	-	-
17	40.45	267.6, 305.3	429.0850	349.00, 285.0973, 253.05, 217.1088	Chrysin 7-*O*-glucuronide	Chry-GluA	[53]	+	+	+
18	40.89	272.3, 311.7	459.0957	283.0610	Oroxylin A 7-*O*-glucuronide (oroxyloside)	Orx-GluA	[50]	+	-	+
19	41.83	270.4, 315.9	445.0802	269.0455, 217.1085, 145.05	Baicalein 6-*O*-glucuronide	Baic-6-GluA	[50]	+	-	+
20	42.30	273.8, 340.3	459.0955	283.0626, 268.0388, 175.0245, 113.0234	Wogonin 7-*O*-glucuronide (wogonoside)	Wog-GluA	[50]	+	-	+
21	43.99	280.0, 358.0	269.0469	241.0515, 225.0562, 197.0607	Norwogonin	Norw	[50]	+	+	-
22	44.51	282.1, 330	299.0575	284.0341, 153.0187	Hispidulin	Hisp	[53,54]	+	+	-
23	44.75	275.5, 322.3	269.0469	251.0364, 145.0500	Baicalein	Baic	[50,52]	+	+	+
24	46.74	268.3, 313.6	283.0625	268.0382, 217.1084, 163.0032, 145.0500	Wogonin	Wog	[50,52]	+	+	-
25	47.00	271.5, 317.8	283.0624	268.0382, 217.1084, 163.0032, 145.0500	Oroxylin A	Orx A	[52]	+	-	-

R_t_—retention time; λ_max_—maximum absorbance; SBR—dried roots of *S. baicalensis*; SBL—dried leaves of *S. baicalensis*; SLL—dried leaves of *S. lateriflora*.

**Table 4:** Concentrations of phenolic compounds in extracts obtained from dried roots of *S. baicalensis* (mg/g DW).

Compound	Extraction Conditions																	
	H_2_O						40% EtOH						70% EtOH					
	21 W		42 W		63 W		21 W		42 W		63 W		21 W		42 W		63 W	
	5 min	10 min	5 min	10 min	5 min	10 min	5 min	10 min	5 min	10 min	5 min	10 min	5 min	10 min	5 min	10 min	5 min	10 min
Chry-Ara-Glu	0.99 ± 0.05	1.29 ± 0.04	1.59 ± 0.03	1.66 ± 0.05	1.49 ± 0.04	1.75 ± 0.03	1.87 ± 0.05	1.99 ± 0.06	2.01 ± 0.04	2.12 ± 0.03	2.04 ± 0.05	2.11 ± 0.02	1.89 ± 0.04	2.18 ± 0.05	1.88 ± 0.03	2.10 ± 0.04	1.89 ± 0.04	1.94 ± 0.05
Chry-Glu-Ara	0.66 ± 0.04	0.85 ± 0.03	1.07 ± 0.05	1.11 ± 0.03	1.01 ± 0.04	1.18 ± 0.04	1.17 ± 0.03	1.23 ± 0.05	1.20 ± 0.03	1.25 ± 0.04	1.24 ± 0.05	0.87 ± 0.04	1.03 ± 0.05	1.35 ± 0.05	1.22 ± 0.04	1.35 ± 0.03	1.23 ± 0.04	1.48 ± 0.05
Baic-7-GluA	0.46 ± 0.02	0.62 ± 0.03	0.54 ± 0.04	0.68 ± 0.03	1.12 ± 0.04	1.22 ± 0.05	42.97 ± 0.11	43.64 ± 0.09	46.94 ± 0.12	48.39 ± 0.10	46.35 ± 0.11	51.85 ± 0.13	43.96 ± 0.12	50.81 ± 0.09	46.83 ± 0.11	51.81 ± 0.12	47.31 ± 0.13	52.66 ± 0.10
Dihbaic-GluA	nd	nd	0.02 ± 0.01	0.03 ± 0.01	0.01 ± 0.01	0.04 ± 0.02	0.06 ± 0.02	0.30 ± 0.03	0.44 ± 0.02	0.43 ± 0.01	0.42 ± 0.02	0.48 ± 0.03	0.48 ± 0.02	0.48 ± 0.02	0.44 ± 0.03	0.49 ± 0.03	0.44 ± 0.02	0.49 ± 0.03
Norw-GluA	0.01 ± 0.01	0.04 ± 0.01	0.02 ± 0.01	0.03 ± 0.01	nd	nd	1.43 ± 0.06	1.87 ± 0.04	2.91 ± 0.05	2.91 ± 0.07	2.70 ± 0.06	3.26 ± 0.05	3.07 ± 0.06	3.17 ± 0.06	2.81 ± 0.05	3.00 ± 0.07	2.85 ± 0.04	2.88 ± 0.05
Hdimf-GluA	nd	nd	nd	nd	nd	nd	0.71 ± 0.03	0.69 ± 0.02	0.84 ± 0.03	0.88 ± 0.03	0.86 ± 0.04	0.91 ± 0.02	0.88 ± 0.02	0.90 ± 0.03	0.80 ± 0.03	0.90 ± 0.02	0.81 ± 0.03	0.85 ± 0.02
Orx-Glu	nd	nd	0.05 ± 0.01	0.07 ± 0.02	0.02 ± 0.01	0.04 ± 0.02	0.28 ± 0.01	0.33 ± 0.02	0.35 ± 0.01	0.32 ± 0.02	0.36 ± 0.03	0.35 ± 0.02	0.37 ± 0.02	0.38 ± 0.01	0.33 ± 0.03	0.35 ± 0.02	0.34 ± 0.02	0.38 ± 0.02
Chry-GluA	nd	nd	nd	nd	nd	nd	0.54 ± 0.02	0.60 ± 0.03	0.81 ± 0.04	0.74 ± 0.03	0.80 ± 0.04	0.83 ± 0.03	0.86 ± 0.04	0.88 ± 0.04	0.78 ± 0.04	0.82 ± 0.05	0.78 ± 0.04	0.81 ± 0.03
Orx-GluA	nd	nd	nd	nd	nd	nd	2.06 ± 0.09	2.56 ± 0.08	3.31 ± 0.05	3.39 ± 0.07	3.34 ± 0.06	3.59 ± 0.04	3.05 ± 0.08	3.62 ± 0.06	3.22 ± 0.07	3.58 ± 0.05	3.26 ± 0.06	3.61 ± 0.04
Baic-6-GluA	nd	nd	nd	nd	nd	nd	0.21 ± 0.01	0.24 ± 0.02	0.27 ± 0.01	0.29 ± 0.02	0.29 ± 0.02	0.31 ± 0.03	0.28 ± 0.02	0.28 ± 0.02	0.25 ± 0.02	0.29 ± 0.03	0.25 ± 0.01	0.28 ± 0.02
Wog-GluA	0.02 ± 0.01	0.04 ± 0.02	0.02 ± 0.01	0.04 ± 0.02	nd	nd	7.19 ± 0.08	7.37 ± 0.06	10.35 ± 0.08	10.42 ± 0.07	10.27 ± 0.09	11.43 ± 0.08	11.05 ± 0.09	11.34 ± 0.07	10.04 ± 0.06	11.42 ± 0.08	10.19 ± 0.08	12.07 ± 0.09
Norw	nd	nd	nd	nd	nd	nd	1.49 ± 0.03	1.53 ± 0.04	0.97 ± 0.03	1.01 ± 0.05	1.06 ± 0.04	0.98 ± 0.04	1.07 ± 0.03	1.11 ± 0.03	0.98 ± 0.04	1.07 ± 0.05	0.96 ± 0.05	0.98 ± 0.04
Hisp	nd	nd	nd	nd	nd	nd	0.45 ± 0.02	0.48 ± 0.03	0.43 ± 0.02	0.35 ± 0.02	0.39 ± 0.03	0.38 ± 0.01	0.46 ± 0.03	0.47 ± 0.02	0.36 ± 0.02	0.39 ± 0.01	0.35 ± 0.03	0.42 ± 0.02
Baic	0.04 ± 0.01	0.05 ± 0.02	0.07 ± 0.02	0.06 ± 0.02	0.06 ± 0.02	0.13 ± 0.01	15.09 ± 0.09	15.50 ± 0.07	15.11 ± 0.08	16.34 ± 0.07	17.50 ± 0.09	15.42 ± 0.08	15.08 ± 0.09	16.29 ± 0.07	15.78 ± 0.08	16.22 ± 0.08	15.66 ± 0.11	17.64 ± 0.09
Wog	nd	nd	nd	nd	nd	nd	4.11 ± 0.05	4.55 ± 0.06	3.97 ± 0.05	3.84 ± 0.06	4.38 ± 0.07	4.22 ± 0.05	3.78 ± 0.04	4.51 ± 0.05	4.04 ± 0.05	4.39 ± 0.06	4.02 ± 0.07	4.59 ± 0.06
Orx A	nd	nd	nd	nd	nd	nd	1.52 ± 0.03	1.71 ± 0.04	1.35 ± 0.03	1.46 ± 0.03	1.64 ± 0.05	1.59 ± 0.04	1.48 ± 0.02	1.54 ± 0.03	1.55 ± 0.04	1.70 ± 0.04	1.54 ± 0.03	1.67 ± 0.04
Total	2.18 ± 0.14	2.89 ± 0.15	3.38 ± 0.18	3.68 ± 0.19	3.71 ± 0.19	4.36 ± 0.17	81.15 ± 0.38	84.59 ± 0.31	91.26 ± 0.25	94.14 ± 0.27	93.64 ± 0.36	98.58 ± 0.37	88.79 ± 0.33	99.31 ± 0.32	91.31 ± 0.27	99.88 ± 0.30	91.88 ± 0.25	102.75 ± 0.31

Results are presented as the mean ±SD from triplicate determinations; nd—not detected; Chry-Ara-Glu—chrysin 6-*C*-arabinoside-8-*C*-glucoside; Chry-Glu-Ara—chrysin 6-*C*-glucoside-8-*C*-arabinoside; Baic-7-GluA—baicalin; Dihbaic-GluA—dihydrobaicalin; Norw-GluA—norwogonoside; Hdimf-GluA—hydroxyldimethoxyflavanone glucuronide; Orx-Glu—oroxylin A 7-*O*-glucoside; Chry-GluA—chrysin 7-*O*-glucuronide; Orx-GluA—oroxyloside; Baic-6-GluA–baicalein 6-*O*-glucuronide; Wog-GluA—wogonoside; Norw—norwogonin; Hisp—hispidulin; Baic—baicalein; Wog—wogonin; Orx A—oroxylin A.

**Table 5:** Concentrations of phenolic compounds in extracts obtained from dried leaves of *S. baicalensis* (mg/g DW).

Compound	Extraction Conditions																	
	H_2_O						40% EtOH						70% EtOH					
	21 W		42 W		63 W		21 W		42 W		63 W		21 W		42 W		63 W	
	5 min	10 min	5 min	10 min	5 min	10 min	5 min	10 min	5 min	10 min	5 min	10 min	5 min	10 min	5 min	10 min	5 min	10 min
Phf-GluA	0.14 ± 0.01	0.19 ± 0.01	0.15 ± 0.03	0.19 ± 0.02	0.09 ± 0.03	0.10 ± 0.02	0.72 ± 0.03	0.74 ± 0.03	0.67 ± 0.04	0.75 ± 0.04	0.69 ± 0.04	0.93 ± 0.04	0.69 ± 0.03	0.86 ± 0.04	0.43 ± 0.03	0.62 ± 0.04	0.79 ± 0.02	1.00 ± 0.03
Phf-GluA iso	0.20 ± 0.01	0.21 ± 0.01	0.24 ± 0.04	0.26 ± 0.05	0.11 ± 0.02	0.10 ± 0.03	0.68 ± 0.04	0.70 ± 0.04	0.63 ± 0.03	0.71 ± 0.04	0.66 ± 0.03	0.88 ± 0.03	0.54 ± 0.04	0.63 ± 0.04	0.70 ± 0.03	1.14 ± 0.05	0.60 ± 0.04	0.78 ± 0.03
Ap-Glu-Ara	0.71 ± 0.01	0.93 ± 0.01	1.12 ± 0.05	1.33 ± 0.04	1.11 ± 0.05	1.37 ± 0.06	1.16 ± 0.04	1.23 ± 0.04	1.28 ± 0.07	1.44 ± 0.06	1.27 ± 0.06	1.76 ± 0.06	1.10 ± 0.07	1.24 ± 0.04	1.43 ± 0.05	1.66 ± 0.05	1.25 ± 0.06	1.63 ± 0.04
IsoCart-GluA	1.39 ± 0.01	1.58 ± 0.01	1.40 ± 0.06	1.54 ± 0.06	1.54 ± 0.05	1.95 ± 0.07	11.12 ± 0.10	11.19 ± 0.09	10.74 ± 0.12	12.28 ± 0.11	11.02 ± 0.10	13.84 ± 0.13	12.98 ± 0.11	15.14 ± 0.12	14.50 ± 0.09	17.38 ± 0.13	13.41 ± 0.11	18.41 ± 0.13
Scu-GluA	1.39 ± 0.01	1.48 ± 0.01	1.16 ± 0.04	1.68 ± 0.06	1.88 ± 0.07	1.91 ± 0.06	7.62 ± 0.09	8.66 ± 0.08	8.55 ± 0.09	9.58 ± 0.11	8.62 ± 0.13	9.95 ± 0.12	6.34 ± 0.08	7.48 ± 0.09	7.70 ± 0.11	9.93 ± 0.12	6.35 ± 0.08	9.53 ± 0.11
Cart-GluA	0.39 ± 0.01	0.44 ± 0.01	0.36 ± 0.03	0.40 ± 0.05	0.29 ± 0.03	0.32 ± 0.03	2.27 ± 0.06	2.39 ± 0.04	2.40 ± 0.05	2.75 ± 0.06	2.44 ± 0.06	3.33 ± 0.08	1.29 ± 0.04	1.07 ± 0.05	1.40 ± 0.07	1.63 ± 0.06	1.22 ± 0.08	1.69 ± 0.04
Ap-GluA	0.42 ± 0.01	0.44 ± 0.01	0.43 ± 0.02	0.54 ± 0.03	0.43 ± 0.04	0.56 ± 0.03	1.15 ± 0.05	1.34 ± 0.04	1.31 ± 0.06	1.47 ± 0.05	1.32 ± 0.05	1.84 ± 0.04	1.11 ± 0.03	1.34 ± 0.05	1.52 ± 0.05	1.72 ± 0.03	1.30 ± 0.04	1.73 ± 0.03
Baic-7-GluA	0.55 ± 0.01	0.71 ± 0.01	0.69 ± 0.03	0.89 ± 0.04	0.78 ± 0.03	0.91 ± 0.04	1.82 ± 0.05	2.34 ± 0.06	2.32 ± 0.07	2.57 ± 0.05	2.45 ± 0.05	3.32 ± 0.04	2.24 ± 0.06	2.54 ± 0.06	2.87 ± 0.07	2.70 ± 0.07	2.59 ± 0.06	3.32 ± 0.05
Hisp-GluA	0.48 ± 0.01	0.63 ± 0.01	0.37 ± 0.04	0.43 ± 0.03	0.28 ± 0.03	0.23 ± 0.04	1.98 ± 0.04	2.49 ± 0.05	2.42 ± 0.06	2.67 ± 0.05	2.48 ± 0.06	3.47 ± 0.06	2.06 ± 0.05	2.49 ± 0.05	2.78 ± 0.04	3.17 ± 0.06	2.44 ± 0.04	3.12 ± 0.04
Norw-GluA	nd	nd	nd	nd	nd	nd	nd	0.85 ± 0.03	0.84 ± 0.03	0.89 ± 0.04	0.85 ± 0.03	1.15 ± 0.03	0.75 ± 0.04	0.92 ± 0.03	1.01 ± 0.05	1.35 ± 0.04	0.88 ± 0.04	1.14 ± 0.03
Chry-GluA	0.09 ± 0.02	0.17 ± 0.03	0.08 ± 0.02	0.08 ± 0.02	0.05 ± 0.01	0.08 ± 0.02	1.55 ± 0.04	1.76 ± 0.01	1.81 ± 0.03	2.02 ± 0.04	1.82 ± 0.05	2.54 ± 0.06	1.69 ± 0.04	1.96 ± 0.03	2.18 ± 0.04	2.13 ± 0.05	1.92 ± 0.05	2.44 ± 0.04
Norw	nd	nd	nd	nd	nd	nd	nd	nd	0.06 ± 0.02	0.06 ± 0.01	0.18 ± 0.03	0.13 ± 0.03	nd	nd	0.30 ± 0.03	0.18 ± 0.04	0.23 ± 0.04	0.35 ± 0.03
Hisp	0.05 ± 0.01	0.07 ± 0.01	nd	nd	nd	nd	0.11 ± 0.02	0.07 ± 0.01	nd	nd	0.10 ± 0.02	0.37 ± 0.04	0.07 ± 0.02	0.07 ± 0.01	0.05 ± 0.01	0.06 ± 0.01	0.08 ± 0.02	0.10 ± 0.02
Baic	0.03 ± 0.01	0.04 ± 0.01	0.05 ± 0.01	0.08 ± 0.02	0.10 ± 0.02	0.12 ± 0.02	0.27 ± 0.02	0.46 ± 0.03	0.22 ± 0.02	0.25 ± 0.02	0.36 ± 0.02	0.37 ± 0.03	0.30 ± 0.02	0.32 ± 0.02	0.36 ± 0.01	0.24 ± 0.02	0.34 ± 0.02	0.44 ± 0.03
Wog	0.10 ± 0.02	0.11 ± 0.02	0.13 ± 0.03	0.21 ± 0.02	0.13 ± 0.02	0.26 ± 0.03	0.30 ± 0.03	0.48 ± 0.03	0.35 ± 0.02	0.36 ± 0.02	0.47 ± 0.03	0.56 ± 0.03	0.63 ± 0.02	0.62 ± 0.03	0.70 ± 0.03	1.15 ± 0.05	0.62 ± 0.03	0.80 ± 0.04
Total	5.94 ± 0.12	7.00 ± 0.13	6.18 ± 0.11	7.63 ± 0.15	6.79 ± 0.17	7.91 ± 0.14	30.75 ± 0.18	34.7 ± 0.19	33.60 ± 0.22	37.80 ± 0.18	34.73 ± 0.21	44.44 ± 0.23	31.79 ± 0.21	36.68 ± 0.19	37.93 ± 0.23	45.06 ± 0.21	33.94 ± 0.22	46.48 ± 0.21

Results are presented as the mean ±SD from triplicate determinations; nd—not detected; Phf-GluA—5,6,7,3′,4′-pentahydroxyflavanon 7-*O*-glucoronide; Ap-Glu-Ara—apigenin 6-*C*-glucoside-8-*C*-arabinoside; IsoCart-GluA—isocarthamidin 7-*O*-glucuronide; Scu-GluA—scutellarin; Cat-GluA—carthamidin 7-O-glucuronide; Ap-GluA—apigenin 7-*O*-glucuronide; Baic-7-GluA—baicalin; Hisp-GluA—hispiduloside; Norw-GluA—norwogonoside; Chry-GluA—chrysin 7-*O*-glucuronide; Norw—norwogonin; Hisp—hispidulin; Baic—baicalein; Wog—wogonin.

**Table 6:** Concentrations of phenolic compounds in extracts obtained from dried leaves of *S. lateriflora* (mg/g DW).

Compound	Extraction Conditions																	
	H_2_O						40% EtOH						70% EtOH					
	21 W		42 W		63 W		21 W		42 W		63 W		21 W		42 W		63 W	
	5 min	10 min	5 min	10 min	5 min	10 min	5 min	10 min	5 min	10 min	5 min	10 min	5 min	10 min	5 min	10 min	5 min	10 min
IsoCart-GluA	0.09 ± 0.02	0.07 ± 0.02	0.08 ± 0.03	0.08 ± 0.02	0.09 ± 0.02	0.13 ± 0.02	0.11 ± 0.03	0.12 ± 0.03	0.13 ± 0.03	0.13 ± 0.04	0.13 ± 0.02	0.11 ± 0.03	0.17 ± 0.03	0.17 ± 0.04	0.17 ± 0.03	0.17 ± 0.03	0.15 ± 0.04	0.18 ± 0.03
Chry-Ara-Glu	0.23 ± 0.03	0.24 ± 0.02	0.28 ± 0.03	0.29 ± 0.03	0.34 ± 0.03	0.37 ± 0.02	0.26 ± 0.02	0.33 ± 0.02	0.30 ± 0.01	0.36 ± 0.03	0.35 ± 0.02	0.29 ± 0.02	0.28 ± 0.01	0.35 ± 0.02	0.28 ± 0.03	0.28 ± 0.02	0.26 ± 0.02	0.31 ± 0.02
Scu-GluA	0.06 ± 0.02	0.03 ± 0.01	nd	nd	nd	nd	0.97 ± 0.04	0.99 ± 0.03	1.08 ± 0.04	1.08 ± 0.03	1.07 ± 0.03	1.09 ± 0.04	1.01 ± 0.03	1.02 ± 0.03	0.96 ± 0.03	0.97 ± 0.03	1.02 ± 0.04	1.16 ± 0.03
Isoscu-GluA	nd	nd	nd	nd	0.02 ± 0.01	0.03 ± 0.01	0.59 ± 0.04	0.61 ± 0.04	0.65 ± 0.04	0.67 ± 0.03	0.66 ± 0.03	0.66 ± 0.02	0.61 ± 0.02	0.64 ± 0.03	0.59 ± 0.05	0.60 ± 0.04	0.63 ± 0.04	0.72 ± 0.03
Ap-GluA	0.19 ± 0.01	0.17 ± 0.02	0.16 ± 0.02	0.18 ± 0.02	0.19 ± 0.03	0.25 ± 0.01	0.19 ± 0.03	0.19 ± 0.02	0.20 ± 0.02	0.19 ± 0.01	0.19 ± 0.02	0.21 ± 0.02	0.24 ± 0.02	0.23 ± 0.03	0.24 ± 0.02	0.25 ± 0.01	0.23 ± 0.02	0.27 ± 0.03
Baic-7-GluA	0.21 ± 0.01	0.37 ± 0.02	0.25 ± 0.03	0.52 ± 0.03	0.48 ± 0.03	0.85 ± 0.03	31.14 ± 0.15	31.46 ± 0.20	33.74 ± 0.16	34.38 ± 0.11	33.67 ± 0.15	34.40 ± 0.16	31.24 ± 0.13	32.44 ± 0.12	30.22 ± 0.16	32.52 ± 0.12	32.35 ± 0.11	36.33 ± 0.14
Dihbaic-GluA	nd	nd	nd	nd	0.02 ± 0.01	0.03 ± 0.01	3.30 ± 0.06	3.54 ± 0.04	3.80 ± 0.04	3.94 ± 0.05	3.79 ± 0.05	3.92 ± 0.04	3.94 ± 0.05	4.06 ± 0.04	3.99 ± 0.06	3.93 ± 0.05	3.76 ± 0.06	4.38 ± 0.05
Hisp-GluA	0.03 ± 0.01	0.04 ± 0.01	0.03 ± 0.01	0.03 ± 0.01	0.04 ± 0.01	0.07 ± 0.02	1.57 ± 0.03	1.59 ± 0.02	1.70 ± 0.04	1.74 ± 0.03	1.71 ± 0.05	1.73 ± 0.03	1.58 ± 0.04	1.64 ± 0.04	1.53 ± 0.05	1.54 ± 0.03	1.63 ± 0.04	1.84 ± 0.04
Norw-GluA	0.18 ± 0.01	0.25 ± 0.012	0.22 ± 0.02	0.28 ± 0.01	0.33 ± 0.02	0.47 ± 0.03	0.63 ± 0.03	1.29 ± 0.04	0.64 ± 0.03	1.41 ± 0.02	1.37 ± 0.03	1.68 ± 0.04	0.60 ± 0.03	1.37 ± 0.03	0.63 ± 0.02	0.63 ± 0.03	0.67 ± 0.02	0.76 ± 0.03
Hdimf-GluA	nd	nd	nd	nd	nd	nd	1.69 ± 0.05	1.69 ± 0.04	1.75 ± 0.06	1.83 ± 0.04	1.76 ± 0.05	1.81 ± 0.04	1.57 ± 0.03	1.71 ± 0.04	1.59 ± 0.03	1.60 ± 0.03	1.69 ± 0.04	1.93 ± 0.03
Chry-GluA	nd	nd	nd	nd	0.02 ± 0.01	0.04 ± 0.02	nd	nd	0.01 ± 0.01	0.03 ± 0.01	0.03 ± 0.01	0.07 ± 0.02	0.02 ± 0.01	0.03 ± 0.01	0.13 ± 0.03	0.14 ± 0.02	0.13 ± 0.01	0.13 ± 0.02
Orx-GluA	nd	nd	0.01 ± 0.01	0.03 ± 0.01	0.02 ± 0.01	0.03 ± 0.01	1.71 ± 0.04	1.71 ± 0.02	1.80 ± 0.03	1.84 ± 0.03	1.79 ± 0.04	1.86 ± 0.04	1.69 ± 0.03	1.81 ± 0.03	1.73 ± 0.04	1.75 ± 0.04	1.80 ± 0.03	2.04 ± 0.04
Baic-6-GluA	nd	nd	0.02 ± 0.01	0.03 ± 0.01	0.04 ± 0.01	0.08 ± 0.02	0.36 ± 0.03	0.35 ± 0.03	0.37 ± 0.02	0.38 ± 0.03	0.37 ± 0.02	0.38 ± 0.02	0.34 ± 0.03	0.36 ± 0.01	0.33 ± 0.02	0.34 ± 0.02	0.36 ± 0.02	0.41 ± 0.03
Wog-GluA	nd	nd	0.01 ± 0.01	0.02 ± 0.01	0.04 ± 0.02	0.08 ± 0.02	1.59 ± 0.03	1.53 ± 0.02	1.65 ± 0.04	1.69 ± 0.03	1.60 ± 0.02	1.70 ± 0.03	1.56 ± 0.03	1.67 ± 0.03	1.59 ± 0.02	1.61 ± 0.03	1.65 ± 0.03	1.90 ± 0.02
Baic	0.03 ± 0.01	0.06 ± 0.02	0.03 ± 0.01	0.03 ± 0.01	0.05 ± 0.02	0.15 ± 0.03	0.48 ± 0.04	0.52 ± 0.02	0.51 ± 0.03	0.59 ± 0.03	0.70 ± 0.02	1.49 ± 0.04	1.49 ± 0.02	1.61 ± 0.03	1.59 ± 0.03	1.60 ± 0.02	1.52 ± 0.02	1.61 ± 0.03
Total	1.03 ± 0.11	1.24 ± 0.13	1.11 ± 0.12	1.50 ± 0.10	1.67 ± 0.14	2.58 ± 0.16	44.60 ± 0.23	45.92 ± 0.21	48.33 ± 0.25	51.15 ± 0.24	49.19 ± 0.18	51.39 ± 0.22	46.35 ± 0.23	49.13 ± 0.19	45.57 ± 0.26	51.32 ± 0.21	47.87 ± 0.25	51.95 ± 0.24

Results are presented as the mean ±SD from triplicate determinations; nd—not detected; IsoCart-GluA—isocarthamidin 7-*O*-glucuronide; Chry-Ara-Glu—chrysin 6-*C*-arabinoside-8-*C*-glucoside; Scu-GluA—scutellarin; Isoscu-GluA—isoscutellarein 7-*O*-glucuronide; Ap-GluA—apigenin 7-*O*-glucuronide; Baic-7-GluA—baicalin; Dihbaic-GluA—dihydrobaicalin; Hisp-GluA—hispiduloside; Norw-GluA—norwogonoside; Hdimf-GluA—hydroxyldimethoxyflavanone glucuronide; Chry-GluA—chrysin 7-*O*-glucuronide; Orx-GluA—oroxyloside; Baic-6-GluA—baicalein 6-*O*-glucuronide; Wog-GluA—wogonoside; Baic—baicalein.

## Data Availability

Data available on request.

## Supplementary Materials

The following supporting information can be downloaded at: https://www.mdpi.com/article/10.3390/molecules28093877/s1. Table S1: The free radical scavenging activity DPPH assay as IC_50_ of optimal extracts from dried roots and leaves of *S. baicalensis* and dried leaves of *S. lateriflora*. Figure S1: Structures of phenolic compounds found in extracts from dried roots and leaves of *S. baicalensis* and dried leaves of *S. lateriflora*. Chemical structures were drawn using ACD/ChemSketch software.

## Sample Availability

Samples of the polyphenol compounds are available from the authors.
