# Supplementary material for: The Influence of Microwave-Assisted Extraction on the Phenolic Compound Profile and Biological Activities of Extracts from Selected Scutellaria Species
Source: Molecules. 2023 May 4;28(9):3877. doi: 10.3390/molecules28093877 (PMC10180449; doi:10.3390/molecules28093877)
Supplement: Supplementary file 1 [file molecules-28-03877-s001.zip › molecules-2363638-supplementary.pdf]

molecules

MDPI

Supplementary Materials

The Influence of Microwave-Assisted Extraction on the Phenolic Compound Profile and Biological Activities of Extracts from Selected *Scutellaria* Species

Joanna Oracz [1,*], Stanisław Kowalski [2], Dorota Żyżelewicz [1,*], Gabriela Kowalska [1], Dorota Gumul [2], Kamila Kulbat-Warycha [1], Justyna Rosicka-Kaczmarek [1], Anna Brzozowska [1], Aleksandra Grzegorczyk [1] and Anna Areczuk [2]

[1] Institute of Food Technology and Analysis, Faculty of Biotechnology and Food Sciences, Lodz University of Technology, 2/22 Stefanowskiego Street, 90-537 Lodz, Poland; kamila.kulbat-warycha@p.lodz.pl (K.K-W.), gabriela.kowalska@p.lodz.pl (G.K.), justyna.rosicka-kaczmarek@p.lodz.pl (J.R-K.), 249773@edu.p.lodz.pl (A.B.), 249776@edu.p.lodz.pl (A.G.)

[2] University of Agriculture in Krakow, Faculty of Food Technology, Department of Carbohydrate Technology and Cereal Processing, Poland, 30-149 Krakow, Balicka Str. 122; rrkowals@cyf-kr.edu.pl (S.K.), rrgumul@cyf-kr.edu.pl (D.G.), anna.areczuk@urk.edu.pl (A.A.)

* Correspondence: joanna.oracz@p.lodz.pl (J.O.); dorota.zyzelewicz@p.lodz.pl (D.Ż.), Tel.: +48 42 631 34 62, Fax: +48 42 631 32 43

Contents

Table S1. The free radical-scavenging activity DPPH assay as IC_{50} of optimal extracts from dried roots and leaves of *S. baicalensis* and dried leaves of *S. lateriflora*.

Figure S1. Structures of phenolic compounds found in extracts from dried roots and leaves of *S. baicalensis* and dried leaves of *S. lateriflora*. Chemical structures were drawn using ACD/ChemSketch software.

Table S1. The free radical-scavenging activity DPPH assay as IC_{50} of optimal extracts from dried roots and leaves of *S. baicalensis* and dried leaves of *S. lateriflora*.

Plant material	Extraction conditions			DPPH (IC_{50}, μg/mL)
	Solvent	Microwave power	Time	
Root of *S. baicalensis*	70% ethanol	63 W	10 min	38.64 ± 0.12
Leaves of *S. baicalensis*				43.41 ± 0.17
Leaves of *S. lateriflora*				37.46 ± 0.13

Results are presented as the mean ±SD from triplicate determinations; SBR—dried roots of *S. baicalensis*; SBL—dried leaves of *S. baicalensis*; SLL—dried leaves of *S. lateriflora*.

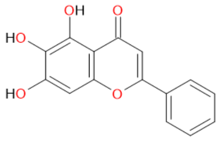

Baicalein (23)

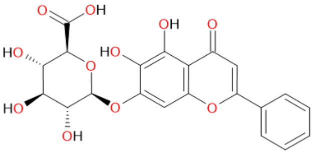

Baicalin (11)

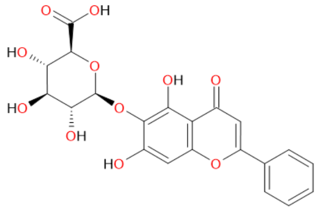

Baicalein 6-*O*-glucuronide (19)

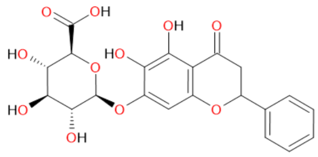

Dihydrobaicalin (12)

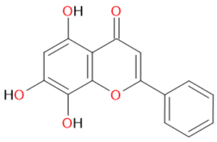

Norwogonin (21)

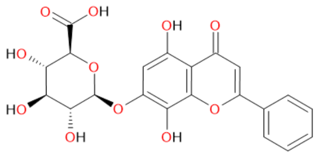

Norwogonoside (14)

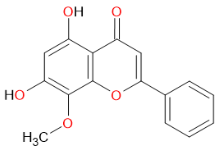

Wogonin (24)

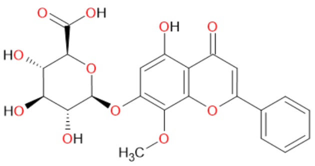

Wogonoside (20)

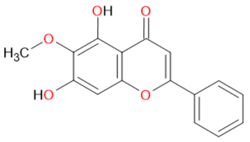

Oroxylin A (25)

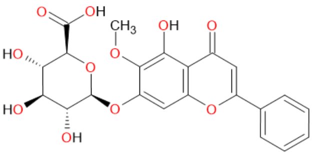

Oroxyloside (18)

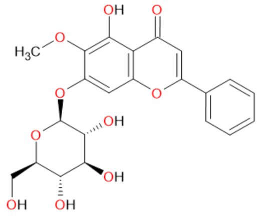

Oroxylin A 7-*O*-glucoside (16)

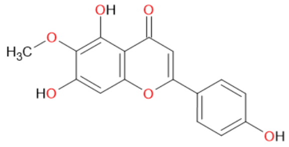

Hispidulin (22)

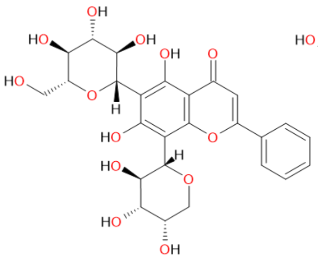

Chrysin 6-*C*-glucoside-8-*C*-arabinoside (8)

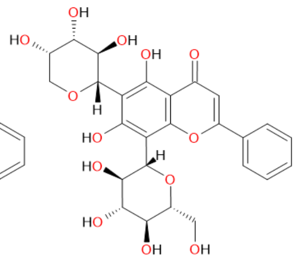

Chrysin 6-*C*-arabinoside-8-*C*-glucoside (5)

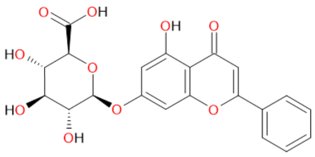

Chrysin 7-O-glucuronide (17)

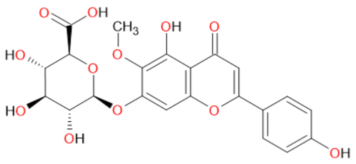

Hispiduloside (13)

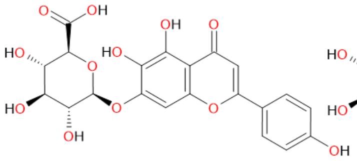

Scutellarin (6)

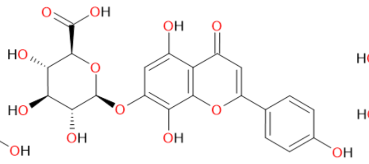

Isoscutellarein 7-*O*-glucuronide (9)

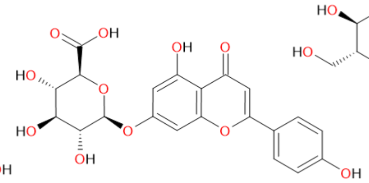

Apigenin 7-O-glucuronide (10)

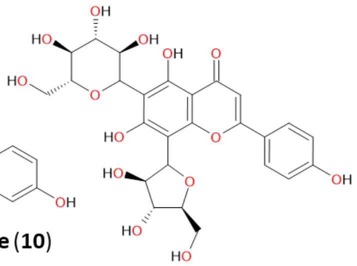

Apigenin 6-*C*-glucoside-8-*C*-arabinoside (3)

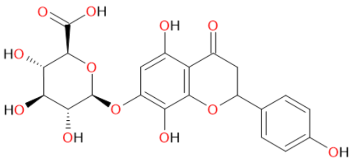

Isocarthamidin 7-*O*-glucuronide (4)

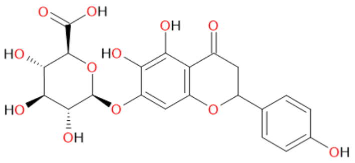

Carthamidin 7-*O*-glucuronide (7)

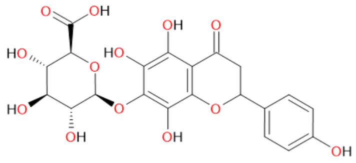

5,6,7,3',4'-Pentahydroxyflavanon 7-*O*-glucoronide(1,2)

Figure S1. Structures of phenolic compounds found in extracts from dried roots and leaves of *S. baicalensis* and dried leaves of *S. lateriflora*. Chemical structures were drawn using ACD/ChemSketch software.
